# Distal and Proximal Influences on Self-Reported Oral Pain and Self-Rated Oral Health Status in Saudi Arabia: Retrospective Study Using a 2017 Nationwide Database

**DOI:** 10.2196/53585

**Published:** 2024-12-20

**Authors:** Naif Abogazalah, Constantin Yiannoutsos, Armando E Soto-Rojas, Naif Bindayeld, Juan F Yepes, Esperanza Angeles Martinez Mier

**Affiliations:** 1 College of Dentistry King Khalid University Abha Saudi Arabia; 2 Fairbanks School of Public Health Indiana University Indianapolis, IN United States; 3 School of Dentistry Indiana University Indianapolis, IN United States; 4 College of Dentistry King Saud University Riyadh Saudi Arabia

**Keywords:** dental health surveys, nationwide database, public health dentistry, path analysis, oral health influences, oral pain, self-rated oral health, cross-sectional study, dental health, dentistry, oral health, self-reported, retrospective study, Saudi Arabia, proximal, distal, adult, children, youth, adolescent, teen, sociodemographic

## Abstract

**Background:**

Oral health significantly influences overall well-being, health care costs, and quality of life. In Saudi Arabia, the burden of oral diseases, such as dental caries and periodontal disease, has increased over recent decades, driven by various lifestyle changes.

**Objective:**

To explore the associations between proximal (direct) and distal (indirect) influences that affect oral pain (OP) and self-rated oral health (SROH) status in the Kingdom of Saudi Arabia (KSA) using an adapted conceptual framework.

**Methods:**

This retrospective cross-sectional study used data from a national health survey conducted in KSA in 2017. The sample included adults (N=29,274), adolescents (N=9910), and children (N=11,653). Sociodemographic data, health characteristics, and access to oral health services were considered distal influences, while frequency and type of dental visits, tooth brushing frequency, smoking, and consumption of sweets and soft drinks were considered proximal influences. Path analysis modeling was used to estimate the direct, indirect, and total effects of proximal and distal influences on OP and SROH status.

**Results:**

The mean age of adult respondents was 42.2 years; adolescents, 20.4 years; and children, 10.58 years. Despite OP reports from 39% of children, 48.5% of adolescents, and 47.1% of adults, over 87% across all groups rated their oral health as good, very good, or excellent. A higher frequency of tooth brushing showed a strong inverse relationship with OP and a positive correlation with SROH (*P*<.001). Frequent dental visits were positively associated with OP and negatively with SROH (*P*<.001). Sweet consumption increased OP in adolescents (β=0.033, *P*=.007) and negatively affected SROH in children (β=–0.086, *P*<.001), adolescents (β=–0.079, *P*<.001), and adults (β=–0.068, *P*<.001). Soft drink consumption, however, was associated with lower OP in adolescents (β=–0.034, *P*=.005) and improved SROH in adolescents (β=0.063, *P*<.001) and adults (β=0.068, *P*<.001). Smoking increased OP in adults (β=0.030, *P*<.001). Distal influences like higher education were directly linked to better SROH (β=0.046, *P*=.003) and less OP (indirectly through tooth brushing, β=–0.004, *P*<.001). For children, high household income correlated with less OP (β=–0.030, *P*=.02), but indirectly increased OP through other pathways (β=0.024, *P*=.003). Lack of access was associated with negative oral health measures (*P*<.001).

**Conclusions:**

Among the KSA population, OP and SROH were directly influenced by many proximal and distal influences that had direct, indirect, or combined influences on OP and SROH status.

## Introduction

Oral health has a major impact on overall health, medical costs, and quality of life. Major oral conditions include dental caries, periodontal disease, and tooth loss. Between 1990 and 2017, the global burden of these conditions increased by 38% [1].

There are reports of an increase in the burden of oral diseases in the Kingdom of Saudi Arabia (KSA) over the last few decades [2]. This increase is likely due to transformations in lifestyle, such as changes in dietary habits, particularly an increase in consumption of sugary foods and tobacco products [3]. Thus, oral health conditions constitute one of the major public health concerns in KSA.

Self-reported oral health status has been used as an important subjective health indicator of oral health care needs and to evaluate the individual’s quality of life [4]. Self-reported information is a cost-effective and time-saving method of data collection. Self-reported oral health can be affected by several factors, such as sociodemographic and socioeconomic factors, cultural values and beliefs, and existing oral health conditions [5].

The Multidimensional Conceptual Model of Oral Health proposed by Gilbert et al [6] states that oral diseases and related tissue damage can result in oral pain (OP) and challenges in daily living that affect self-rated oral health (SROH) status. OP can cause difficulties in chewing and sleep disturbances [7]. In addition, it can affect school and work attendance, causing a loss of a significant number of study and working hours per year [8]. Because of these concerns, OP is frequently incorporated into national health surveys. A 1989 report from the United States reported that 14.5% of adults experienced OP during the past 6 months [9], while in the United Kingdom, 28% of adults were reported to experience difficulty from OP during the past year in 1998 [10].

SROH status serves as a valuable indicator of general oral health status [6]. It is considered a comprehensive index reflecting various dimensions of oral health, including functional, psychological, and social impacts on overall well-being [11]. It has been linked to clinical oral health status, such as dental caries, tooth mobility, and tooth loss [4]. Furthermore, SROH has been found to predict future oral health outcomes, as seen in longitudinal studies assessing maternal SROH and their children's caries experience in adulthood [12].

Distal and proximal influences play significant roles in shaping oral health outcomes such as OP and SROH. Proximal influences such as oral health-related behaviors and the use of oral health services directly impact oral health [13]. On the other hand, distal influences encompass broader determinants such as socioeconomic status and access to care determinants, which also have a substantial influence on oral health outcomes [14]. Understanding the interplay between distal and proximal influences is essential for addressing oral health status among populations and developing effective interventions to improve oral health outcomes across diverse populations.

**Figure 1:**
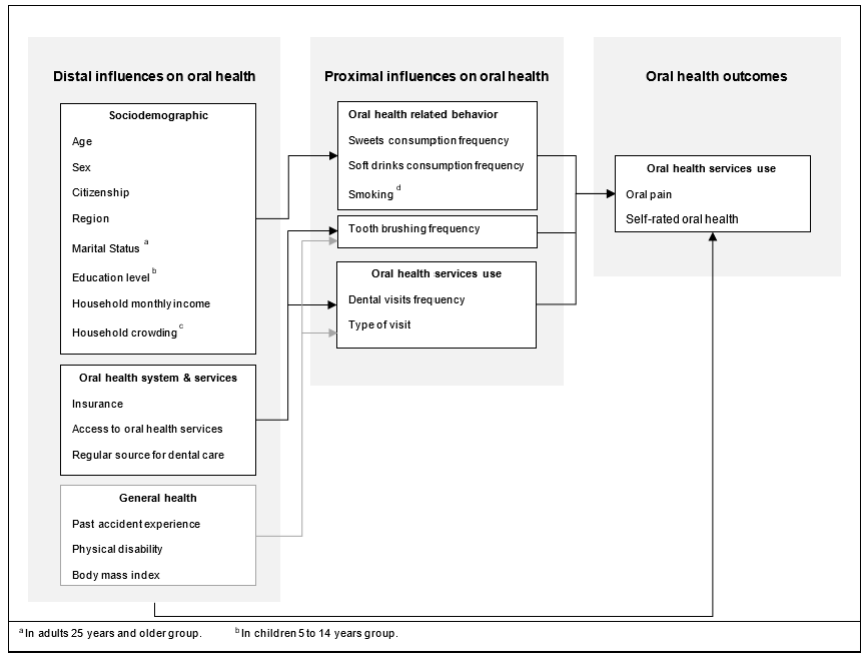
Conceptual framework for distal and proximal influences on self-reported oral pain and oral health status in Saudi Arabia.

The use of conceptual frameworks for understanding determinants in oral health research can serve as a coherent map to guide researchers when inquiring about oral health conditions. Conceptual frameworks can also help researchers to include multiple factors that may explain an outcome and aid in designing statistical analyses [15]. The objective of this study was to explore how proximal and distal influences on oral health are related to both OP experience and SROH status among KSA residents by using data from a national demographic and health survey (DHS) that was conducted in 2017 in KSA. A conceptual framework was developed (Figure 1) to guide the analysis.

## Methods

### Data Source

The original data collection was approved by the institutional review board (IRB) of the Ministry of Health of the Kingdom of Saudi Arabia (Central IRB Log #: 2019-0131M). No additional IRB approval was needed for the secondary analysis, as it qualifies under Exemption 4 of US federal regulations [45 CFR 46.104(d)(4)] due to the use of existing, nonidentifiable data. The authors have permission to use the data, which was collected with participant consent. Data analysis was conducted at the Indiana University School of Dentistry, the Department of Biostatistics at the Indiana University School of Medicine, and the Richard M. Fairbanks School of Public Health, Indianapolis. The Human Research Protection Program from the Office of Research Compliance at Indiana University determined that this secondary analysis does not require further IRB review (Protocol #: 1808825963). Neither the study principal investigator nor key personnel had any financial conflict of interest concerning this research. The data were available at the office of the Directorate of Primary Health Care Centers (Ministry of Health, Headquarters, Riyadh, KSA). The Ministry of Health used a probability multistage stratified random sampling for the DHS. Details of the sampling procedure were published previously [16]. Briefly, house-to-house visits were conducted to interview the head of a family or an eligible representative and other specific family members between February 12, 2017, and May 23, 2017. Participants answered questions related to demographic, environmental, and health-related topics. The data were received in SPSS (IBM Corp) software format, and an analysis file was created, which comprised selected variables of interest. In total, 3 parallel analyses were performed based on the age of the respondent: children, 5-14 years; adolescents, 15-24 years; and adults, ≥25 years (details are given in [14]). The analysis was done at the Biostatistics Department, Indiana University School of Medicine, Indianapolis, Indiana, United States (IRB protocol number: 1808825963).

### Development of the Model

Based on existing models, a multi-level conceptual framework was developed for oral health influences in KSA (Figure 1) on self-reported OP and SROH status among KSA residents. Constructs from the Multidimensional Conceptual Model of Oral Health proposed by Gilbert et al [6] and the World Health Organization Model for Oral Health Surveillance [17] were adapted by expanding the concept of proximal (direct) influences on oral health—such as diet and oral hygiene—to include distal (indirect) influences such as socioeconomic determinants.

### Selection of Endogenous and Exogenous Variables

Model variables were selected after a careful review of the literature, identifying those that were both available in the survey and previously reported to influence OP and SROH status. The model variables were classified as exogenous and endogenous. Exogenous variables are those that are not affected by other variables in the model (the distal or indirect influences), while endogenous variables are affected by other variables in the model, such as proximal influences and outcome variables. In total, 13 exogenous variables were included for the adult group, and 11 and 12 exogenous variables were included for the adolescent and children groups, respectively. In total, 8 endogenous variables were included for the adult group and 7 endogenous variables were included for the adolescent and children's groups, respectively. The variables are listed in (Table 1). Exogenous variables (distal influence variables) included the age [18] of participants as a continuous variable and gender [19] as a binary variable (males or females). Citizenship [20] status was coded as a binary variable: citizens and noncitizens. Geographic regions [2] were classified into the East, West, and Central versus the North and South. Marital status was dichotomized into currently married and not married (≥25 years only). Completed education level [21] was categorized as primary, intermediate, high school, intermediate diploma, and college or higher education. In total, 5 levels of household monthly income [22] were included: lower class income (3800 Riyals or less), marginal middle-class income (3801-7699 Riyals), basic middle-class income (7700-22,900 Riyals), upper middle-class income (22,901-38,200 Riyals), and upper-class income (>38,200 Riyals). Household crowding was calculated by dividing the number of family members by the number of sleeping rooms. The responses were then grouped into 4 levels: <1, 1-2, 2-3, and >3 persons per room. Past accident experience and physical disability were assessed as binary no or yes responses. BMI was dichotomized as normal (BMI=18.5-24.9) and abnormal (BMI <18.5 and >24.9). Health insurance was expressed as a binary variable of insured versus not insured. Access to oral health services [23] in the year prior to the survey was dichotomized into “no or I do not know and yes” responses. The source of dental care was dichotomized into a government versus private clinic.

**Table 1:** Weighted and non-weighted descriptive statistics for model variables by age group from the 2017 National Demographic and Health Survey conducted by the Ministry of Health of Saudi Arabia.

Variable			5-14 years						15-24 years							≥25 years											
			N		%		Wt.%		N		%		Wt.%		N					%			Wt.%				
**Sex**																											
	Male	5813		49.9		50.9		4630		46.7		52.2		12,802					43.7			61.2					
	Female	5840		50.1		49.1		5280		53.3		47.8		16,472					56.3			38.8					
**Citizenship**																											
	Citizen	10603		91.0		75.8		9064		91.5		79.6		26,225					89.6			53.2					
	Noncitizen	1050		9.0		24.2		846		8.5		20.4		3049					10.4			46.8					
**Region**																											
	East, west, and central	8349		71.6		75.9		7032		71.0		75.2		21,634					73.9			79.3					
	North and south	3304		28.4		24.1		2878		29.0		24.8		7640					26.1			20.7					
**Marital status (≥25 years only)**																											
	Married	—^a^		—		—		—		—		—		11,537					90.3			87.9					
	Not married	—		—		—		—		—		—		1243					9.7			12.1					
**Education (≥25 years only)**																											
	Primary school education	—		—		—		—		—		—		3167					14.2			14.8					
	Intermediate school education	—		—		—		—		—		—		7465					33.4			38.9					
	High school education	—		—		—		—		—		—		6364					28.4			24.8					
	Intermediate Diploma	—		—		—		—		—		—		1282					5.7			5.0					
	College or higher education	—		—		—		—		—		—		4092					18.3			16.5					
**Monthly household income**																											
	≤3800 Riyals	2134		37.8		40.4		1733		38.0		39.6		3599					21.6			25.3					
	3801–7699 Riyals	1258		22.3		21.9		1222		26.8		26.9		4190					25.1			29.7					
	7700–22,900 Riyals	2188		38.7		36.5		1552		34.0		32.3		8084					48.5			39.6					
	22,901–38,200 Riyals	40		0.7		0.6		32		0.7		0.7		694					4.2			4.8					
	>38,200 Riyals	29		0.5		0.5		24		0.5		0.6		109					0.7			0.6					
**Household crowding (5–14 years only)**																											
	≤1 person/room	528		12.4		13.2		—		—		—		—					—			—					
	1-2 person/room	1967		46.1		45.5		—		—		—		—					—			—					
	2-3 person/room	1153		27.0		26.6		—		—		—		—					—			—					
	>3 person/room	622		14.6		14.6		—		—		—		—					—			—					
**Accident**																											
	No	10,656		95.7		95.5		8826		93.4		93.0					26,168	94.8			94.4						
	Yes	483		4.3		4.5		624		6.6		7.0					1449	5.2			5.6						
**Disability**																											
	No	11,000		98.7		98.7		9237		98.4		98.4					27,112	98.3			98.2						
	Yes	147		1.3		1.3		147		1.6		1.6					480	1.7			1.8						
**BMI (kg/m^2^)**																											
	Abnormal (<18.5 and >24.9)	5432		61.2		61.3		4940		60.4		61.6					14,643	59.6			63.2						
	Normal (18.5–24.9)	3439		38.8		38.7		3233		39.6		38.4					9925	40.4			36.8						
**Health insurance**																											
	No	3477		78.4		70.7		2643		76.0		70.8					9396	73.7			59.9						
	Yes	957		21.6		29.3		836		24.0		29.2					3346	26.3			40.1						
**Access to health care**																											
	Available/I don't know	8133		76.5		78.6		6725		74.9		76.5					18,666	70.5			76.5						
	Not available	2498		23.5		21.4		2251		25.1		23.5					7808	29.5			23.5						
**Source of care**																											
	Government clinic	4146		81.5		76.3		4737		77.1		73.3					14,224	75.3			62.9						
	Private dental clinic or other clinic	941		18.5		23.7		1403		22.9		26.7					4663	24.7			37.1						
**Sweets consumption frequency**																											
	I don't eat at all	813		7.3		7.5		1795		19.0		19.4		5467					19.7			20.5					
	Many times per month	4468		40.0		39.8		4217		44.7		44.4		12,379					44.6			44.9					
	Once per week	1441		12.9		13.7		1340		14.2		14.6		3924					14.2			14.0					
	Many times per week	2723		24.4		24.1		1451		15.4		15.1		4059					14.6			14.1					
	Once per day	1173		10.5		10.2		437		4.6		4.7		1344					4.8			4.5					
	Many times per day	550		4.9		4.6		188		2.0		1.8		556					2.0			2.0					
**Soft drinks consumption**																											
	I don't drink at all	3476		31.3		31.5		3180		33.7		35.0		9297					33.5			37.1					
	Many times per month	3897		35.1		34.9		3341		35.4		34.5		9948					35.9			34.6					
	Once per week	1313		11.8		11.8		1184		12.5		12.3		3239					11.7			11.7					
	Many times per week	1794		16.1		16.3		1275		13.5		13.4		3795					13.7			12.4					
	Once per day	538		4.8		4.8		366		3.9		3.8		1070					3.9			3.2					
	Many times per day	91		0.8		0.7		100		1.1		1.0		389					1.4			1.1					
**Smoking (≥25 years only)**																											
	No	—		—		—		—		—		—		25,937					91.5			93.0					
	Yes	—		—		—		—		—		—		2404					8.5			7.0					
**Tooth brushing frequency**																											
	Never	1097		10.8		10.7		1026		11.7		11.1		3047					11.8			11.6					
	I clean my teeth somedays but not daily	3234		31.7		31.5		2185		24.9		24.9		6482					25.1			24.5					
	Once weekly	718		7.0		7.1		716		8.2		8.4		2139					8.3			9.0					
	Many times per week	1385		13.6		13.2		1023		11.7		11.6		2902					11.2			11.0					
	Once daily	2558		25.1		25.3		2321		26.5		27.1		6909					26.8			27.3					
	Twice or more daily	1201		11.8		12.3		1495		17.1		16.9		4341					16.8			16.5					
**Dental visits frequency**																											
	Never visited a dentist or don’t know or don’t remember	1434		14.0		5.5		1219		13.7		13.4		6562					5.0			13.4					
	Not visited a dentist in the past year	4068		39.8		8.9		3223		36.2		35.7		9900					8.2			36.0					
	Once	2489		24.4		41.8		2096		23.6		23.9		6206					37.8			24.5					
	More than once	2227		21.8		23.2		2356		26.5		27.0		6606					23.7			26.1					
**Type of visit**																											
	For a complaint	5395		91.6		91.0		5233		93.8		93.8		15,175					94.2			94.6					
	Routine examination and treatment	497		8.4		9.0		346		6.2		6.2		932					5.8			5.4					
**Oral pain**																											
	Never felt	3825		37.8		39.8		2936		33.7		32.2		8779					34.0			32.5					
	Rarely	2145		21.2		21.2		1658		19.0		19.3		4971					19.3			20.4					
	Sometimes	2548		25.2		23.8		2275		26.1		26.9		6809					26.4			26.3					
	Many times	1603		15.8		15.2		1849		21.2		21.6		5260					20.4			20.8					
**Self-rated oral health status**																											
	Bad	122		1.2		1.1		173		2.0		2.1		548					2.2			2.4					
	Acceptable	627		6.3		6.0		865		10.1		10.8		2372					9.4			10.5					
	Good	2756		27.7		27.6		2581		30.3		30.6		7624					30.2			32.8					
	Very good	3998		40.1		39.9		3278		38.4		38.3		9539					37.8			36.2					
	Excellent	2462		24.7		25.4		1635		19.2		18.2		5141					20.4			18.1					

^a^Not applicable.

Endogenous variables (proximal influences and outcome variables) included the frequency of consuming sweets [24], individuals responded to the following question “How often do you eat sweets?” as “I don't eat at all, many times per month, once per week, many times per week, once per day, many times per day.” For soft drinks consumption frequency [25], “How often do you drink soft drinks?” responses were “I don't drink at all, many times per month, once per week, many times per week, once per day, many times per day.” For smoking status, [26] yes or no responses to the question “Do you smoke?” Frequency of tooth brushing [27,28] had 6 levels: “I have never cleaned my teeth, I clean my teeth some days but not daily, once weekly, many times per week, once daily, twice or more daily.” The frequency of dental visits was determined as from response to the question “How many times have you visited a dentist in the past year?” Valid answers include “never visited a dentist/I do not know or do not remember, did not visit the dentist in the past year, once, more than once.” For the type of visit, responses were dichotomized into visits for a complaint versus visits for routine examination and treatment [22]. For OP [6], the question was phrased as: “How many times during the past year have you felt pain in your teeth?” There were 4 levels of response: “never felt, rarely, sometimes, many times.” For SROH [6], participants were asked, “How would you describe the health of your teeth and gums?” Responses were “bad, acceptable, good, very good, excellent.”

### Data Analyses

SPSS (IBM Corp) was used to perform descriptive analysis for the model variables (Table 1). R software (R Foundation for Statistical Computing) was used to perform the path analysis considering the sample weights. Missing values were not replaced or imputed in this analysis. The first step was to assess the multivariate normality of endogenous variables. Both skewness and kurtosis statistics confirmed that endogenous variables did not follow a multivariate normal distribution (*P*<.05). Owing to the presence of non-normal and missing data, full-information maximum likelihood estimation to perform path analysis available in the lavaan package (version 0.6.12) was used [29]. Robust standard errors (Huber-White) and scaled test statistics were calculated [30]. The software estimated the direct effect, as hypothesized in the model in (Figure 1), of each oral health influence, as well as the indirect effect for each exogenous variable on OP and SROH status through a path mediated by each proximal influence on oral health (Figure 1). For example, the effect of sex on OP was mediated by the frequency of tooth brushing. The total indirect effects on OP and SROH status reflected the effect of the path between each exogenous variable via all proximal influences on oral health. The total effects comprised the sum of the total indirect and direct effects of each distal influence on OP and SROH status.

A separate model was estimated for each age group—children 5–14 years, adolescents 15–24 years, and adults ≥25 years. Model fit was evaluated using the robust comparative fit index>0.9, robust Tucker-Lewis index>0.9, robust root mean-square-error of approximation <0.08, and robust standardized root-mean-square residual<0.08 [31].

## Results

The conceptual model (Figure 1) states that OP and SROH status are directly influenced by distal and proximal influences on oral health. Furthermore, OP and SROH status are indirectly influenced by distal influences via all proximal influences except past accident experience, physical disability, and BMI, where they were indirectly influenced by OP and SROH status via only dental visit frequency, type of visit, and frequency of tooth brushing (Figure 1).

The final analysis included 29,274 adults ≥25 years of age (mean 42.2, SD 12.97), 9910 adolescents aged 15–24 (mean 20.4, SD 2.98) years, and 11,653 children aged 5 and 14 (mean 10.58, SD 2.84) years. Complete descriptive statistics are published elsewhere [16]. Table 1 presents a summary of the weighted and non-weighted estimates.

Despite 39% children, 48.5% adolescents, and 47.1% adults reporting OP in the past year, 92.9% children, 87.1% of adolescents, and 87.1% adults reported good, very good, or excellent SROH status, respectively.

The model goodness-of-fit measures showed an acceptable fit to the data, meeting the recommended values for the fit statistics (Table 2) [31].

**Table 2:** Model fit indices values.

Index	Values for the model of each age group						
	Non-weighted				Weighted		
	5-14 years	15-24 years	25+ years	5-14 years		15-24 years	25+ years
Robust comparative fit index (RCFI)	0.964	0.927	0.920	0.966		0.971	0.958
Robust Tucker–Lewis index (RTLI)	0.889	0.813	0.806	0.908		0.919	0.884
Robust root-mean-square error of approximation (RRMSEA)	0.029	0.036	0.035	0.027		0.024	0.028
Robust standardized root-mean-square residual (RSRMR)	0.020	0.027	0.029	0.031		0.020	0.028

### Proximal Influences on Oral Health Effects

A higher tooth brushing frequency was strongly associated with less OP and positive SROH status in all groups. In contrast, a higher number of dental visits was associated with more OP and less favorable SROH status in all age groups. Routine examination and treatment were linked to less OP in all age groups and better SROH status in the adult and adolescent groups. Consumption of sweets was linked to greater OP in adolescents (β=0.033, *P*=.007) and negative SROH status in children (β=–0.086, *P*<.001), adolescents (β=–0.079, *P*<.001), and adults (β=–0.068, *P*<.001). Soft drinks were linked to lower OP in the adolescent group (β=–0.034, *P*=.005). Higher consumption of soft drinks was associated positively with SROH status (β=0.063, *P*<.001) in the adolescent and adult groups (β=0.068, *P*<.001). Smoking was associated with more OP (β=0.030, *P*<.001) in the adult group (Table 3).

**Table 3:** Direct effects of the distal and proximal influences on oral pain and self-rated oral health status based on conceptual framework (Figure 1). Data from the 2017 National Demographic and Health Survey conducted by the Ministry of Health of Saudi Arabia.

Pathway			5-14 years										15-24 years										≥25 years							
			β^a^		SE		*P* value		95% CI			β			SE		*P* value		95% CI			β			SE		*P* value		95% CI	
**Oral pain**																														
	Age	0.009		0.011		.426		–0.013		0.030	0.006			0.010		.54		–0.014		0.026	–0.010			0.010		.33		–0.029		0.010
	Sex	0.017		0.009		.06		–0.001		0.035	0.003			0.011		.76		–0.018		0.024	0.008			0.011		.43		–0.012		0.029
	Citizenship	–0.081		0.014		*<.001* ^d^		–0.109		–0.053	–0.004			0.016		.81		–0.035		0.027	–0.015			0.013		.23		–0.040		0.010
	Region	–0.006		0.009		.465		–0.024		0.011	–0.018			0.010		.07		–0.038		0.002	–0.046			0.013		*<.001*		–0.071		–0.021
	Marital status (≥25 years only)	—^e^		—		—		—		—	—			—		—		—		—	0.022			0.015		.13		–0.006		0.051
	Education (≥25 years only)	—		—		—		—		—	—			—		—		—		—	–0.006			0.014		.67		–0.033		0.021
	Monthly household income	–0.030		0.013		*.02*		–0.055		–0.004	0.010			0.015		.49		–0.019		0.039	–0.021			0.015		.16		–0.050		0.008
	Household crowding (5-14 years only)	0.002		0.016		.89		–0.029		0.033	—			—		—		—		—	—			—		—		—		—
	Health insurance	–0.036		0.017		*.033*		–0.068		–0.003	0.051			0.018		*.004*		0.016		0.085	0.042			0.015		*<.001*		0.012		0.072
	Access to health care	0.195		0.010		*<.001*		0.175		0.215	0.009			0.010		.34		–0.010		0.029	0.015			0.010		.11		–0.003		0.034
	Source of care	0.053		0.016		*.001*		0.022		0.083	0.012			0.014		.41		–0.016		0.040	–0.001			0.016		.94		–0.032		0.030
	Accident	0.051		0.010		*<.001*		0.032		0.070	0.028			0.009		*.002*		0.010		0.046	0.007			0.013		.60		–0.019		0.032
	Disability	0.012		0.009		.16		–0.005		0.030	0.008			0.009		.38		–0.009		0.025	0.027			0.008		*.001*		0.011		0.042
	BMI (kg/m^2^)	0.015		0.011		.18		–0.007		0.038	–0.033			0.011		*.003*		–0.055		–0.011	–0.013			0.011		.27		–0.035		0.010
	Sweets consumption frequency	0.007		0.010		.52		–0.014		0.027	0.033			0.012		*.007*		0.009		0.057	0.019			0.012		.10		–0.004		0.041
	Soft drinks consumption	0.021		0.011		.058		–0.001		0.043	–0.034			0.012		*.005*		–0.058		–0.01	–0.023			0.012		.06		–0.047		0.001
	Smoking (≥25 years only)	—		—		—		—		—	—			—		—		—		—	0.045			0.012		*<.001*		0.021		0.069
	Tooth brushing frequency	–0.038		0.010		*<.001*		–0.057		–0.018	–0.098			0.01		*<.001*		–0.118		–0.078	–0.079			0.011		*<.001*		–0.101		–0.058
	Dental visits frequency	0.510		0.012		*<.001*		0.486		0.534	0.614			0.01		*<.001*		0.594		0.633	0.577			0.012		*<.001*		0.554		0.600
	Type of visit	–0.064		0.014		*<.001*		–0.090		–0.037	–0.063			0.015		*<.001*		–0.092		–0.034	–0.088			0.016		*<.001*		–0.120		–0.056
**Self-rated oral health status**																														
	Age	–0.044		0.013		*.001*		–0.069		–0.018	–0.033			0.013		*.008*		–0.058		–0.009	0.013			0.011		.22		–0.008		0.035
	Sex	–0.030		0.011		*.009*		–0.052		–0.007	0.023			0.013		.09		–0.003		0.048	–0.020			0.012		.11		–0.045		0.004
	Citizenship	0.067		0.018		*<.001*		0.032		0.102	0.013			0.020		.53		–0.026		0.051	–0.050			0.015		*.001*		–0.080		–0.020
	Region	–0.080		0.012		*<.001*		–0.103		–0.057	–0.014			0.013		.26		–0.039		0.011	–0.008			0.014		.55		–0.035		0.019
	Marital status (≥25 years only)	—		—		—		—		—	—			—		—		—		—	–0.009			0.019		.62		–0.046		0.027
	Education (≥25 years only)	—		—		—		—		—	—			—		—		—		—	0.046			0.015		*.003*		0.016		0.077
	Monthly household income	0.073		0.016		*<.001*		0.042		0.104	0.018			0.018		.31		–0.017		0.053	–0.028			0.018		.11		–0.063		0.006
	Household crowding (5-14 years only)	–0.045		0.020		*.02*		–0.084		–0.006	—			—		—		—		—	—			—		—		—		—
	Health insurance	–0.014		0.020		.49		–0.053		0.025	–0.160			0.021		*<.001*		–0.201		–0.119	–0.135			0.018		*<.001*		–0.171		–0.099
	Access to health care	–0.096		0.013		*<.001*		–0.122		–0.071	0.045			0.013		*.001*		0.019		0.070	0.016			0.011		.14		–0.005		0.038
	Source of care	–0.136		0.018		*<.001*		–0.172		–0.100	–0.001			0.017		.96		–0.035		0.033	0.019			0.018		.29		–0.017		0.055
	Accident	–0.033		0.012		*.007*		–0.057		–0.009	–0.011			0.014		.44		–0.038		0.016	–0.043			0.011		*<.001*		–0.065		–0.022
	Disability	–0.016		0.010		.12		–0.036		0.004	–0.078			0.018		*<.001*		–0.112		–0.044	–0.049			0.018		*.006*		–0.085		–0.014
	BMI	0.051		0.013		*<.001*		0.025		0.077	0.093			0.013		*<.001*		0.067		0.118	0.067			0.013		*<.001*		0.042		0.093
	Sweets consumption frequency	–0.086		0.013		*<.001*		–0.112		–0.061	–0.079			0.015		*<.001*		–0.109		–0.05	–0.065			0.014		*<.001*		–0.092		–0.039
	Soft drinks consumption	0.022		0.013		.10		–0.004		0.048	0.063			0.015		*<.001*		0.035		0.092	0.068			0.014		*<.001*		0.040		0.096
	Smoking (≥25 years only)	—		—		—		—		—	—			—		—		—		—	–0.012			0.015		.40		–0.042		0.017
	Tooth brushing frequency	0.129		0.013		*<.001*		0.104		0.154	0.162			0.014		*<.001*		0.135		0.189	0.158			0.014		*<.001*		0.131		0.186
	Dental visits frequency	–0.130		0.014		*<.001*		–0.157		–0.104	–0.143			0.014		*<.001*		–0.17		–0.116	–0.132			0.015		*<.001*		–0.161		–0.104
	Type of visit	0.021		0.015		.18		–0.010		0.051	0.046			0.015		*.002*		0.017		0.075	0.047			0.015		*.001*		0.018		0.075

^a^β: standardized regression weights.

^b^Significant pathways in italic font.

^c^Not applicable.

### Distal Influences on Oral Health Effects

Tables 3-5 illustrate the direct, total indirect, and total effects, respectively, of oral health influences on OP and SROH status. Tables S1, S2, and S3 in Multimedia Appendix 1 show the direct effects of the distal influences on the proximal influences and the indirect effects of each distal influence on both OP and SROH status via each proximal influence.

**Table 4:** Total indirect effects for the distal influences on oral pain and self-rated oral health status based on conceptual framework (Figure 1). Data from the 2017 National Demographic and Health Survey conducted by the Ministry of Health of Saudi Arabia.

Pathway		5–14 years						15–24 years						≥25 years				
		β^a^	SE	*P* value	95% CI		β		SE	*P* value	95% CI		β		SE	*P* value	95% CI	
**Oral pain**																		
	Age	0.062	0.007	*<.001* ^b^	0.048	0.077	0.015		0.008	.06	–0.001	0.030	–0.002		0.007	.83	–0.016	0.013
	Sex	0.000	0.006	.97	–0.011	0.011	–0.003		0.009	.73	–0.020	0.014	–0.016		0.008	*.04*	–0.032	–0.001
	Citizenship	–0.004	0.009	.66	–0.022	0.014	0.026		0.014	.06	–0.001	0.053	0.023		0.009	*.02*	0.004	0.041
	Region	0.027	0.006	*<.001*	0.016	0.037	–0.014		0.009	.11	–0.032	0.003	0.000		0.009	.96	–0.017	0.018
	Marital status (≥25 years only)	—^c^	—	—	—	—	—		—	—	—	—	0.003		0.014	.86	–0.025	0.030
	Education (≥25 years only)	—	—	—	—	—	—		—	—	—	—	0.010		0.011	.37	–0.012	0.031
	Monthly household monthly income	0.024	0.008	*.003*	0.008	0.040	–0.006		0.012	.60	–0.029	0.017	0.010		0.012	.43	–0.014	0.033
	Household crowding (5-14 years only)	–0.010	0.009	.30	–0.028	0.009	—		—	—	—	—	—		—	—	—	—
	Health insurance	–0.023	0.010	*.03*	–0.043	–0.003	–0.024		0.017	.17	–0.058	0.010	–0.019		0.013	.13	–0.043	0.006
	Access to health care	0.215	0.007	*<.001*	0.202	0.227	–0.012		0.009	.17	–0.028	0.005	–0.010		0.007	.15	–0.023	0.003
	Source of care	–0.014	0.010	.17	–0.033	0.006	–0.016		0.012	.20	–0.039	0.008	–0.018		0.012	.14	–0.041	0.006
	Accident	0.038	0.006	*<.001*	0.026	0.050	0.014		0.009	.10	–0.003	0.031	–0.016		0.009	.06	–0.033	0.001
	Disability	0.007	0.006	.21	–0.004	0.019	–0.004		0.009	.66	–0.023	0.014	0.004		0.010	.68	–0.016	0.024
	BMI (kg/m^2^)	0.015	0.007	*.03*	0.001	0.028	–0.014		0.009	.12	–0.032	0.004	–0.020		0.008	*.02*	–0.036	–0.004
**Self-rated oral health status**																		
	Age	0.000	0.005	.97	–0.009	0.009	–0.006		0.003	.07	–0.012	0.000	0.001		0.003	.62	–0.004	0.006
	Sex	0.006	0.003	*.04*	0.000	0.011	0.001		0.003	.78	–0.005	0.007	0.006		0.003	*.03*	0.001	0.012
	Citizenship	0.005	0.004	.20	–0.002	0.012	–0.004		0.005	.42	–0.014	0.006	–0.005		0.004	.20	–0.012	0.002
	Region	–0.019	0.003	*<.001*	–0.024	–0.014	0.003		0.004	.36	–0.004	0.011	–0.003		0.005	.53	–0.013	0.006
	Marital status (≥25 years only)	—	—	—	—	—	—		—	—	—	—	–0.003		0.005	.58	–0.013	0.007
	Education (≥25 years only)	—	—	—	—	—	—		—	—	—	—	0.004		0.004	.30	–0.004	0.012
	Monthly household income	–0.003	0.003	.42	–0.010	0.004	0.000		0.005	.98	–0.009	0.009	0.000		0.005	.97	–0.009	0.009
	Household crowding (5-14 years only)	0.003	0.004	.50	–0.005	0.010	—		—	—	—	—	—		—	—	—	—
	Health insurance	0.003	0.004	.53	–0.006	0.011	0.007		0.007	.28	–0.006	0.021	–0.012		0.006	*.04*	–0.023	0.000
	Access to health care	–0.068	0.006	*<.001*	–0.080	–0.056	0.005		0.003	.14	–0.002	0.012	0.009		0.003	*.002*	0.003	0.014
	Source of care	0.012	0.005	*.009*	0.003	0.021	0.006		0.005	.18	–0.003	0.015	0.010		0.004	*.03*	0.001	0.018
	Accident	–0.007	0.002	*.003*	–0.012	–0.003	–0.003		0.003	.36	–0.010	0.004	0.008		0.003	*.01*	0.002	0.014
	Disability	–0.001	0.002	.77	–0.005	0.004	0.001		0.003	.74	–0.005	0.008	0.000		0.003	.96	–0.006	0.006
	BMI	–0.004	0.003	.10	–0.010	0.001	0.006		0.003	.09	–0.001	0.013	0.004		0.003	.19	–0.002	0.010

^a^β: standardized regression weights.

^b^Significant pathways in italic font.

^c^Not applicable.

**Table 5:** Total effects for the distal influences on oral pain and self-rated oral health status based on conceptual framework (Figure 1). Data from the 2017 National Demographic and Health Survey conducted by the Ministry of Health of Saudi Arabia.

Pathway			5–14 years										15–24 years										≥25 years							
			β^a^		SE		*P* value		95% CI			β			SE		*P* value		95% CI			β			SE		*P* value		95% CI	
**Oral pain**																														
	Age	0.071		0.012		*<.001* ^b^		0.047		0.095	0.021			0.013		.10		–0.004		0.046	–0.011			0.011		.31		–0.033		0.010
	Sex	0.017		0.011		.11		–0.004		0.038	0.000			0.013		.98		–0.026		0.026	–0.008			0.012		.52		–0.032		0.016
	Citizenship	–0.085		0.017		*<.001*		–0.119		–0.052	0.022			0.021		.27		–0.018		0.063	0.008			0.015		.61		–0.022		0.037
	Region	0.020		0.011		.06		–0.001		0.041	–0.033			0.013		*.01*		–0.058		–0.008	–0.045			0.013		*<.001*		–0.070		–0.021
	Marital status (≥25 years only)	—		—		—		—		—	—			—		—		—		—	0.025			0.021		.23		–0.015		0.065
	Education (≥25 years only)	—		—		—		—		—	—			—		—		—		—	0.004			0.017		.82		–0.029		0.036
	Monthly household income	–0.006		0.016		.72		–0.037		0.025	0.004			0.019		.83		–0.032		0.040	–0.011			0.018		.52		–0.046		0.023
	Household crowding (5-14 years only)	–0.008		0.018		.68		–0.043		0.028	—			—		—		—		—	—			—		—		—		—
	Health insurance	–0.058		0.020		*.003*		–0.097		–0.020	0.027			0.024		.26		–0.020		0.073	0.023			0.018		.20		–0.013		0.059
	Access to health care	0.410		0.010		*<.001*		0.390		0.430	–0.002			0.013		.86		–0.028		0.023	0.006			0.011		.61		–0.016		0.028
	Source of care	0.039		0.018		*.03*		0.003		0.074	–0.004			0.018		.83		–0.039		0.032	–0.019			0.018		.30		–0.055		0.017
	Accident	0.089		0.012		*<.001*		0.067		0.112	0.042			0.013		*.001*		0.017		0.067	–0.009			0.014		.51		–0.037		0.018
	Disability	0.020		0.011		.07		–0.002		0.042	0.004			0.012		.77		–0.021		0.028	0.031			0.013		*.02*		0.005		0.057
	BMI	0.030		0.013		*.02*		0.004		0.056	–0.047			0.014		*.001*		–0.074		–0.020	–0.032			0.014		*.02*		–0.059		–0.006
**Self-rated oral health status**																														
	Age	–0.044		0.013		*.001*		–0.069		–0.018	–0.039			0.013		*.002*		–0.063		–0.014	0.015			0.011		.19		–0.007		0.037
	Sex	–0.024		0.012		*.04*		–0.047		–0.002	0.023			0.013		.08		–0.003		0.050	–0.014			0.013		.29		–0.039		0.011
	Citizenship	0.072		0.018		*<.001*		0.037		0.107	0.008			0.020		.67		–0.030		0.047	–0.055			0.016		*<.001*		–0.085		–0.024
	Region	–0.099		0.012		*<.001*		–0.121		–0.076	–0.011			0.013		.39		–0.036		0.014	–0.011			0.013		.40		–0.037		0.015
	Marital status (25+ only)	—		—		—		—		—	—			—		—		—		—	–0.012			0.019		.53		–0.050		0.026
	Education (≥25 years only)	—		—		—		—		—	—			—		—		—		—	0.050			0.016		*.002*		0.019		0.082
	Monthly household income	0.070		0.016		*<.001*		0.038		0.101	0.018			0.018		.31		–0.017		0.054	–0.028			0.019		.14		–0.064		0.009
	Household crowding (5-14 years only)	–0.043		0.020		*.03*		–0.081		–0.005	—			—		—		—		—	—			—		—		—		—
	Health insurance	–0.011		0.020		.58		–0.050		0.028	–0.152			0.022		*<.001*		–0.195		–0.109	–0.146			0.018		*<.001*		–0.182		–0.110
	Access to health care	–0.164		0.011		*<.001*		–0.187		–0.142	0.050			0.013		*<.001*		0.024		0.075	0.025			0.011		*.03*		0.003		0.047
	Source of care	–0.124		0.018		*<.001*		–0.160		–0.088	0.005			0.018		.77		–0.029		0.040	0.029			0.019		.12		–0.007		0.065
	Accident	–0.040		0.012		*.001*		–0.064		–0.016	–0.014			0.013		.29		–0.039		0.012	–0.036			0.012		*.002*		–0.059		–0.013
	Disability	–0.017		0.011		.14		–0.039		0.005	–0.077			0.016		*<.001*		–0.109		–0.045	–0.050			0.018		*.005*		–0.085		–0.015
	BMI	0.047		0.013		*<.001*		0.021		0.073	0.099			0.013		*<.001*		0.072		0.125	0.071			0.014		*<.001*		0.045		0.098

^a^β: standardized regression weights.

^b^Significant pathways in italic font.

^c^Not applicable.

#### Age and Sex

There was a negative direct effect between age and SROH (Table 3) in both the children (β=–0.044, *P*=.001) and adolescent (β=–0.033, *P*=.008) groups. An indirect positive effect (β=0.062, *P*<.001) was found between age and OP in the children group (Table 4). Total effect (Table 5) of age was detected in the children group for both OP (positive relation) and SROH status (negative relation).

Among female children, a negative direct effect (β=–0.030, *P*=.009) was found with SROH (Table 3). Total indirect effect showed a negative association between female adults and OP (β=–0.016, *P*=.04; Table 4). This total indirect effect was mediated by dental visits and tooth brushing frequency, as a positive direct link was found between female sex and tooth brushing frequency in both the child (β=0.073, *P*<.001) and adult groups (β=0.026, *P*=.04; Tables S1 and S3 in Multimedia Appendix 1). Also, the total indirect effect revealed a positive association between female children (β=0.006, *P*=.04) and adults (β=0.006, *P*=.03) with SROH (Table 4). However, total effect showed a negative association between female children and SROH (β=–0.024, *P*=.04; Table 5).

#### Citizenship, Regions, and Education Levels

Among non-Saudi citizens, the direct (β=–0.081, *P*<.001) and total (β=–0.085, *P*<.001) effects showed a negative association with OP in the children’s age group but a positive association in the adult group (β=0.023, *P*=.02) through the total indirect effect.

Adolescents and adults from the north and south regions were linked to less OP than those from the east, west, and central regions through the total (β=–0.033, *P*=.01 and β=–0.045, *P*<.001) effects pathways (Table 5). However, OP was positively linked to children from the southern and northern regions through the total indirect pathway (β=0.027, *P*<.001).

Higher education level was associated with positive SROH status directly (β=0.046, *P*=.003) and through the total direct effect pathway (β=0.050, *P*=.002). Furthermore, higher education was linked with greater tooth brushing frequency and less OP indirectly via tooth brushing frequency (β=–0.004, *P*<.001) and positive SROH status (β=0.008, *P*<.001) (Table S3 in Multimedia Appendix 1).

#### Monthly Household Income and Insurance

In the children group, higher household income was associated with less OP through the direct pathway (β=–0.030, *P*=.02), but higher income was positively associated with greater OP through the total indirect pathway (β=0.024, *P*=.003). In addition, higher income was associated with less OP (β=–0.002, *P*=.03) and a positive SROH status (β=0.006, *P*=.007) when mediated by tooth brushing frequency (Table S1 in Multimedia Appendix 1).

In the children group, having health insurance was associated with less OP through direct (β=–0.36, *P*=.03), total indirect (β=–0.023, *P*=.03), and total effect pathways (β=–0.058, *P*=.003). On the other hand, insurance was associated positively with OP through the direct pathway in both adolescents (β=0.051, *P*=.004) and adults (β=0.042, *P*<.001). Moreover, in adults, insurance was linked to less OP indirectly via dental visit frequency (β=–0.028, *P*=.01).

#### Access to Oral Health Services

In the children group, lack of access to oral health care was linked to more OP and negative SROH status through direct, total indirect, and total effects (Tables 2-4).

Private clinic visits as the regular source of dental care were associated with more OP and worse SROH status in the children group through the direct (for OP: β=0.053, *P*=.001 and for SROH status: β=–0.136, *P*<.001, respectively) and through total effect pathways (for OP: β=0.039, *P*=.03; for SROH status: β=–0.124, *P*<.001). However, private clinic visits as the regular source of dental care were associated with better SROH status through a total indirect effect (β=0.012, *P*=.009). Private clinic as the regular source of dental care was associated with less OP and better SROH status in the children group through the indirect effect via tooth brushing frequency (for OP: β=–0.005, *P*=.003; for SROH status: β=0.017, *P*<.001).

#### Past Accident Experience and Physical Disability

In the adult group, past accident experience was negatively associated with SROH status through the direct (β=–0.043, *P*<.001) and total effects pathways (β=–0.036, *P*=.002) and positively associated with SROH status through the total indirect pathway (β=0.008, *P*=.01).

Physical disability was linked to OP and negative SROH status in the child group through the total-effects pathway (Table 4). In the adult group, physical disability was associated with greater OP and worse SROH status through the direct and total effect pathways (Tables 2 and 4).

#### BMI

Normal BMI was linked to better SROH status through the direct pathway (β=0.051, *P*<.001) and through the total effect pathway (β=0.047, *P*<.001). In the adolescent group, normal BMI was linked to less OP and better SROH status through the direct and total effects pathways (Tables 2 and 4). In the adult group, normal BMI was linked to less OP through the total indirect and total effect pathways (Tables 3 and 4).

## Discussion

### Principal Findings

The findings of this study partially support the adapted conceptual framework for distal and proximal influences on self-reported OP and SROH status in Saudi Arabian residents (Figure 1). All proximal influences were associated with OP, except sweets and soft drink consumption in the children and adult groups. Unexpectedly, the consumption of soft drinks was associated with less OP in the adolescent group, although OP was positively associated with greater consumption of sweets. Moreover, all proximal influences were associated with SROH status except soft drink consumption and type of dental visit in the children’s age group and smoking in the adult group. In addition, greater consumption of sweets and a higher number of dental visits were negatively associated with SROH status in the adolescent and children groups, but soft drink consumption was positively linked to SROH status in the adolescent and adult groups. Regarding distal influences, the majority of them showed an association with both OP and SROH status through the direct, indirect, and total effect pathways.

This study indicated that the prevalence of OP in Saudi Arabia is high. Data from 20 different countries in a meta-analysis illustrated that OP prevalence in children and adolescents was 36.2% [32]. This is almost half of the prevalence found in this study in similar age groups (60.2% for children and 67.8% for adolescents in KSA). Furthermore, the findings from this study differed from National Health and Nutrition Examination Survey (NHANES) 2015-2018 data [33] on adult US residents where OP was associated with education and income level, while in KSA it was not.

The findings from this study indicated that dental visit frequency was positively associated with OP and negatively associated with SROH status, while routine visits were associated with less pain and better SROH status in all age groups. It would be expected that a higher frequency of dental visits would be associated with better oral health; however, it appears that Saudi residents visit the dentist mainly when they have a complaint, such as OP, and do not normally visit the dentist for a routine check-up. A similar trend has been previously reported in national [34] and some subnational studies in Saudi Arabia [35-37]. The lack of interest in routine oral examinations and treatment visits (9.0% of children, 6.2% of adolescents, and 5.4% of adults reported routine dental visits) can be explained by the habitually optimistic view of the Saudi population about their oral health, as 92.9% of children, 87.1% of adolescents, and adults self-rated their oral health as good to excellent despite roughly two-thirds of them having reportedly experienced OP during the previous 6 months. Infrequent routine visits and optimistic SROH status combined with less frequent tooth brushing is a critical finding of this study because the conclusion of these combined observations is that Saudi residents do not recognize risk factors that can adversely affect their oral health. This could be due to the cultures and beliefs in KSA, which are reinforced by the family and community [38]. Oral health officials and policy makers should consider raising public awareness about the importance of oral hygiene and routine dental clinic visits to improve oral health in the general population.

This study had several important limitations. Its cross-sectional nature, like all similar DHS studies, limits the ability to investigate distal and proximal influences on OP and SROH status over time, hindering the assessment of causal relationships between the predictive factors considered and the study outcome. On the other hand, this study is the first to assess the association between a number of predictive factors and oral health outcomes using a conceptual framework to guide the analysis. To our knowledge, no longitudinal study has measured changes in oral health status in Saudi Arabia, which is needed to determine oral health risk factors specific to this country’s population.

Another major limitation in this study is the subjectivity of a self-reported survey over objective clinical evaluation, which may introduce response bias such as recall bias [39] in the responses (such as dental visit frequency in the past year) or social desirability bias [40] (such as not revealing smoking status). Such biases may have influenced the response to the question related to the consumption of soft drinks, particularly in the adolescent group. Adolescents who consume more soft drinks might underreport their OP due to a perception that admitting to pain could lead to restrictions on their soft drink consumption by parents or guardians [41], which in turn may have resulted in the apparent association of increased soft drink consumption with lower OP and better SROH status, which goes against clear evidence of an association between higher soft drink consumption and negative oral health outcomes in a number of cross-sectional and longitudinal studies [42]. Furthermore, self-reported OP and SROH status may not reflect exact oral health clinical status because they express a person’s perception of their OP and oral health, which can be influenced by psychosocial and cultural factors. For example, NHANES 2003-2004 data from the United States showed that Latinos reported better SROH status than White individuals, while Latino individuals had more oral disease and lower access to and use of dental care [43]. Although using SROH indicators over a clinical measurement may have introduced some reporting bias, it is still a convenient, cost-effective, and expedited method to assess oral health status at a national level with a large sample and has shown a positive association with clinical oral health status [20]. Self-reported oral health measures have been used in many countries and national surveys, such as NHANES [33,43].

Despite these limitations, this study has a number of strengths, including the use of data from the 2017 KSA DHS, which applied a random sampling design with a large sample size, over a wide geographic distribution, and broad age range, which ensured the representative nature of the findings to the entire KSA, a heterogeneous country characterized by areas of very high income within a developing country. The second advantage is the use of the conceptual framework to guide the analysis, which enabled the use of path analysis. This is a preferred approach to delineate complex relationships, including both the direct and indirect effects of numerous predictive factors, over traditional multiple regression methods. This enabled testing the direct and indirect effects of different oral health influences on SROH status and OP. In this regard, the conceptual framework in this study is a significant contribution to the understanding of oral health influences in Saudi Arabia. However, the inclusion of other important oral health influences, such as coping skills and social support constructs, could have increased the explanatory power of the study [44].

### Conclusions

Although OP is prevalent among Saudi residents, they still have a positive view of their oral health. Frequent tooth brushing, routine dental visits, and reduced sweet consumption are associated with less OP and better SROH. However, more frequent dental visits seem to address complaints rather than preventive care. Future research should investigate why residents have a positive perception of oral health despite high levels of OP and negative outcomes.

## Appendix

Multimedia Appendix 1 Additional material.

## Abbreviations

DHS: demographic and health survey
IRB: institutional review board
KSA: Kingdom of Saudi Arabia
NHANES: National Health and Nutrition Examination Survey
OP: oral pain
SROH: self-rated oral health

## Data Availability

The datasets generated during and/or analyzed during this study are not publicly available due to Indiana’s University agreement with the Ministry of Health of Saudi Arabia, but can be available from the office of the Directorate of Primary Health Care Centers (Ministry of Health Headquarters, Riyadh, Kingdom of Saudi Arabia).
