# Supplementary material for: Distal and Proximal Influences on Self-Reported Oral Pain and Self-Rated Oral Health Status in Saudi Arabia: Retrospective Study Using a 2017 Nationwide Database
Source: JMIR Public Health Surveill. 2024 Dec 20;10:e53585. doi: 10.2196/53585 (PMC11699488; doi:10.2196/53585)
Supplement: Multimedia Appendix 1 [file publichealth_v10i1e53585_app1.docx]

**Supplemental Materials**

**Table S1. Direct and Indirect Effects on Oral Pain and Self Rated Oral Health Status in Saudi Arabia for 5–14 year olds**

|  | Pathway | β | SE | *p*-value | 95% CI | |
| --- | --- | --- | --- | --- | --- | --- |
| **Age** | | | | | | |
|  | Direct |  |  |  |  |  |
| Dental visits frequency |  | 0.104 | 0.012 | **<0.001** | 0.080 | 0.129 |
| Type of visit |  | -0.116 | 0.018 | **<0.001** | -0.151 | -0.081 |
| Tooth brushing frequency |  | 0.001 | 0.013 | 0.930 | -0.024 | 0.027 |
| Sweets consumption frequency |  | -0.147 | 0.012 | **<0.001** | -0.170 | -0.123 |
| Soft drinks consumption |  | 0.135 | 0.012 | **<0.001** | 0.112 | 0.159 |
|  |  |  |  |  |  |  |
| Oral Pain | Indirect via Dental visits frequency | 0.053 | 0.006 | **<0.001** | 0.041 | 0.066 |
|  | Indirect via Type of visit | 0.007 | 0.002 | **<0.001** | 0.004 | 0.011 |
|  | Indirect via Tooth brushing frequency | 0.000 | 0.000 | 0.930 | -0.001 | 0.001 |
|  | Indirect via Sweets consumption | -0.001 | 0.002 | 0.518 | -0.004 | 0.002 |
|  | Indirect via Soft drinks consumption | 0.003 | 0.002 | 0.060 | 0.000 | 0.006 |
|  |  |  |  |  |  |  |
| Self rated oral health status | Indirect via Dental visits frequency | -0.014 | 0.002 | **<0.001** | -0.018 | -0.009 |
|  | Indirect via Type of visit | -0.002 | 0.002 | 0.196 | -0.006 | 0.001 |
|  | Indirect via Tooth brushing frequency | 0.000 | 0.002 | 0.930 | -0.003 | 0.003 |
|  | Indirect via Sweets consumption | 0.013 | 0.002 | **<0.001** | 0.008 | 0.017 |
|  | Indirect via Soft drinks consumption | 0.003 | 0.002 | 0.103 | -0.001 | 0.007 |
| **Sex** |  |  |  |  |  |  |
|  | Direct |  |  |  |  |  |
| Dental visits frequency |  | 0.011 | 0.010 | 0.287 | -0.009 | 0.031 |
| Type of visit |  | 0.015 | 0.017 | 0.361 | -0.018 | 0.049 |
| Tooth brushing frequency |  | 0.073 | 0.012 | **<0.001** | 0.050 | 0.096 |
| Sweets consumption |  | 0.010 | 0.011 | 0.350 | -0.011 | 0.032 |
| Soft drinks consumption |  | -0.085 | 0.011 | **<0.001** | -0.107 | -0.063 |
|  |  |  |  |  |  |  |
| Oral Pain | Indirect via Dental visits frequency | 0.006 | 0.005 | 0.287 | -0.005 | 0.016 |
|  | Indirect via Type of visit | -0.001 | 0.001 | 0.371 | -0.003 | 0.001 |
|  | Indirect via Tooth brushing frequency | -0.003 | 0.001 | **0.001** | -0.004 | -0.001 |
|  | Indirect via Sweets consumption | 0.000 | 0.000 | 0.602 | 0.000 | 0.000 |
|  | Indirect via Soft drinks consumption | -0.002 | 0.001 | 0.069 | -0.004 | 0.000 |
|  |  |  |  |  |  |  |
| Self rated oral health status | Indirect via Dental visits frequency | -0.001 | 0.001 | 0.288 | -0.004 | 0.001 |
|  | Indirect via Type of visit | 0.000 | 0.000 | 0.411 | 0.000 | 0.001 |
|  | Indirect via Tooth brushing frequency | 0.009 | 0.002 | **<0.001** | 0.006 | 0.013 |
|  | Indirect via Sweets consumption | -0.001 | 0.001 | 0.355 | -0.003 | 0.001 |
|  | Indirect via Soft drinks consumption | -0.002 | 0.001 | 0.107 | -0.004 | 0.000 |
|  |  |  |  |  |  |  |
| Citizenship | | | | | | |
|  | Direct | -0.004 | 0.017 | 0.805 | -0.037 | 0.029 |
| Dental visits frequency |  | 0.009 | 0.028 | 0.757 | -0.046 | 0.064 |
| Type of visit |  | 0.003 | 0.019 | 0.890 | -0.035 | 0.041 |
| Tooth brushing frequency |  | -0.054 | 0.018 | **0.003** | -0.089 | -0.018 |
| Sweets consumption |  | -0.039 | 0.018 | **0.031** | -0.075 | -0.004 |
| Soft drinks consumption |  |  |  |  |  |  |
|  |  |  |  |  |  |  |
| Oral Pain | Indirect via Dental visits frequency | -0.002 | 0.009 | 0.805 | -0.019 | 0.015 |
|  | Indirect via Type of visit | -0.001 | 0.002 | 0.757 | -0.004 | 0.003 |
|  | Indirect via Tooth brushing frequency | 0.000 | 0.001 | 0.890 | -0.002 | 0.001 |
|  | Indirect via Sweets consumption | 0.000 | 0.001 | 0.524 | -0.001 | 0.001 |
|  | Indirect via Soft drinks consumption | -0.001 | 0.001 | 0.142 | -0.002 | 0.000 |
|  |  |  |  |  |  |  |
| Self rated oral health status | Indirect via Dental visits frequency | 0.001 | 0.002 | 0.805 | -0.004 | 0.005 |
|  | Indirect via Type of visit | 0.000 | 0.001 | 0.755 | -0.001 | 0.001 |
|  | Indirect via Tooth brushing frequency | 0.000 | 0.003 | 0.890 | -0.005 | 0.005 |
|  | Indirect via Sweets consumption | 0.005 | 0.002 | **0.008** | 0.001 | 0.008 |
|  | Indirect via Soft drinks consumption | -0.001 | 0.001 | 0.202 | -0.002 | 0.000 |
|  |  |  |  |  |  |  |
| Region | | | | | | |
|  | Direct |  |  |  |  |  |
| Dental visits frequency |  | 0.039 | 0.010 | **<0.001** | 0.019 | 0.059 |
| Type of visit |  | -0.034 | 0.012 | **0.005** | -0.057 | -0.010 |
| Tooth brushing frequency |  | -0.065 | 0.011 | **<0.001** | -0.088 | -0.043 |
| Sweets consumption |  | 0.072 | 0.012 | **<0.001** | 0.048 | 0.096 |
| Soft drinks consumption |  | 0.082 | 0.012 | **<0.001** | 0.059 | 0.106 |
|  |  |  |  |  |  |  |
| Oral Pain | Indirect via Dental visits frequency | 0.020 | 0.005 | **<0.001** | 0.010 | 0.030 |
|  | Indirect via Type of visit | 0.002 | 0.001 | **0.011** | 0.000 | 0.004 |
|  | Indirect via Tooth brushing frequency | 0.002 | 0.001 | **0.002** | 0.001 | 0.004 |
|  | Indirect via Sweets consumption | 0.000 | 0.001 | 0.520 | -0.001 | 0.002 |
|  | Indirect via Soft drinks consumption | 0.002 | 0.001 | 0.072 | 0.000 | 0.004 |
|  |  |  |  |  |  |  |
| Self rated oral health status | Indirect via Dental visits frequency | -0.005 | 0.001 | **<0.001** | -0.008 | -0.002 |
|  | Indirect via Type of visit | -0.001 | 0.001 | 0.180 | -0.002 | 0.000 |
|  | Indirect via Tooth brushing frequency | -0.008 | 0.002 | **<0.001** | -0.012 | -0.005 |
|  | Indirect via Sweets consumption | -0.006 | 0.001 | **<0.001** | -0.009 | -0.003 |
|  | Indirect via Soft drinks consumption | 0.002 | 0.001 | 0.108 | 0.000 | 0.004 |
|  |  |  |  |  |  |  |
| Income | | | | | | |
|  | Direct |  |  |  |  |  |
| Dental visits frequency |  | 0.047 | 0.015 | **0.003** | 0.016 | 0.077 |
| Type of visit |  | -0.035 | 0.023 | 0.122 | -0.079 | 0.009 |
| Tooth brushing frequency |  | 0.047 | 0.017 | **0.006** | 0.014 | 0.079 |
| Sweets consumption |  | 0.020 | 0.017 | 0.220 | -0.012 | 0.053 |
| Soft drinks consumption |  | -0.012 | 0.017 | 0.499 | -0.045 | 0.022 |
|  |  |  |  |  |  |  |
| Oral Pain | Indirect via Dental visits frequency | 0.024 | 0.008 | **0.003** | 0.008 | 0.039 |
|  | Indirect via Type of visit | 0.002 | 0.001 | 0.137 | -0.001 | 0.005 |
|  | Indirect via Tooth brushing frequency | -0.002 | 0.001 | **0.025** | -0.003 | 0.000 |
|  | Indirect via Sweets consumption | 0.000 | 0.000 | 0.567 | 0.000 | 0.001 |
|  | Indirect via Soft drinks consumption | 0.000 | 0.000 | 0.515 | -0.001 | 0.000 |
|  |  |  |  |  |  |  |
| Self rated oral health status | Indirect via Dental visits frequency | -0.006 | 0.002 | **0.004** | -0.010 | -0.002 |
|  | Indirect via Type of visit | -0.001 | 0.001 | 0.266 | -0.002 | 0.001 |
|  | Indirect via Tooth brushing frequency | 0.006 | 0.002 | **0.007** | 0.002 | 0.010 |
|  | Indirect via Sweets consumption | -0.002 | 0.001 | 0.227 | -0.005 | 0.001 |
|  | Indirect via Soft drinks consumption | 0.000 | 0.000 | 0.534 | -0.001 | 0.001 |
|  |  |  |  |  |  |  |
| Household crowding | | | | | | |
|  | Direct |  |  |  |  |  |
| Dental visits frequency |  | -0.020 | 0.018 | 0.246 | -0.055 | 0.014 |
| Type of visit |  | -0.003 | 0.030 | 0.932 | -0.060 | 0.055 |
| Tooth brushing frequency |  | -0.019 | 0.019 | 0.329 | -0.056 | 0.019 |
| Sweets consumption |  | -0.026 | 0.019 | 0.160 | -0.063 | 0.010 |
| Soft drinks consumption |  | 0.003 | 0.019 | 0.864 | -0.034 | 0.041 |
|  |  |  |  |  |  |  |
| Oral Pain | Indirect via Dental visits frequency | -0.010 | 0.009 | 0.247 | -0.028 | 0.007 |
|  | Indirect via Type of visit | 0.000 | 0.002 | 0.932 | -0.004 | 0.004 |
|  | Indirect via Tooth brushing frequency | 0.001 | 0.001 | 0.347 | -0.001 | 0.002 |
|  | Indirect via Sweets consumption | 0.000 | 0.000 | 0.554 | -0.001 | 0.000 |
|  | Indirect via Soft drinks consumption | 0.000 | 0.000 | 0.865 | -0.001 | 0.001 |
|  |  |  |  |  |  |  |
| Self rated oral health status | Indirect via Dental visits frequency | 0.003 | 0.002 | 0.254 | -0.002 | 0.007 |
|  | Indirect via Type of visit | 0.000 | 0.001 | 0.931 | -0.001 | 0.001 |
|  | Indirect via Tooth brushing frequency | -0.002 | 0.002 | 0.327 | -0.007 | 0.002 |
|  | Indirect via Sweets consumption | 0.002 | 0.002 | 0.177 | -0.001 | 0.006 |
|  | Indirect via Soft drinks consumption | 0.000 | 0.000 | 0.866 | -0.001 | 0.001 |
|  |  |  |  |  |  |  |
| Accident | | | | | | |
|  | Direct |  |  |  |  |  |
| Dental visits frequency |  | 0.076 | 0.012 | **<0.001** | 0.053 | 0.098 |
| Type of visit |  | -0.007 | 0.012 | 0.550 | -0.031 | 0.016 |
| Tooth brushing frequency |  | 0.022 | 0.012 | 0.059 | -0.001 | 0.044 |
|  |  |  |  |  |  |  |
| Oral Pain | Indirect via Dental visits frequency | 0.039 | 0.006 | **<0.001** | 0.027 | 0.050 |
|  | Indirect via Type of visit | 0.000 | 0.001 | 0.552 | -0.001 | 0.002 |
|  | Indirect via Tooth brushing frequency | -0.001 | 0.000 | 0.089 | -0.002 | 0.000 |
|  |  |  |  |  |  |  |
| Self rated oral health status | Indirect via Dental visits frequency | -0.010 | 0.002 | **<0.001** | -0.014 | -0.006 |
|  | Indirect via Type of visit | 0.000 | 0.000 | 0.585 | -0.001 | 0.000 |
|  | Indirect via Tooth brushing frequency | 0.003 | 0.002 | 0.063 | 0.000 | 0.006 |
|  |  |  |  |  |  |  |
| Disability | | | | | | |
|  | Direct |  |  |  |  |  |
| Dental visits frequency |  | 0.014 | 0.011 | 0.211 | -0.008 | 0.036 |
| Type of visit |  | -0.010 | 0.010 | 0.331 | -0.029 | 0.010 |
| Tooth brushing frequency |  | 0.011 | 0.011 | 0.348 | -0.012 | 0.033 |
|  |  |  |  |  |  |  |
| Oral Pain | Indirect via Dental visits frequency | 0.007 | 0.006 | 0.211 | -0.004 | 0.019 |
|  | Indirect via Type of visit | 0.001 | 0.001 | 0.342 | -0.001 | 0.002 |
|  | Indirect via Tooth brushing frequency | 0.000 | 0.000 | 0.366 | -0.001 | 0.000 |
|  |  |  |  |  |  |  |
| Self rated oral health status | Indirect via Dental visits frequency | -0.002 | 0.001 | 0.212 | -0.005 | 0.001 |
|  | Indirect via Type of visit | 0.000 | 0.000 | 0.435 | -0.001 | 0.000 |
|  | Indirect via Tooth brushing frequency | 0.001 | 0.001 | 0.351 | -0.002 | 0.004 |
|  |  |  |  |  |  |  |
| Body Mass Index | | | | | | |
|  | Direct |  |  |  |  |  |
| Dental visits frequency |  | 0.028 | 0.013 | **0.029** | 0.003 | 0.053 |
| Type of visit |  | 0.000 | 0.019 | 0.986 | -0.037 | 0.038 |
| Tooth brushing frequency |  | -0.006 | 0.014 | 0.677 | -0.033 | 0.021 |
|  |  |  |  |  |  |  |
| Oral Pain | Indirect via Dental visits frequency | 0.014 | 0.007 | **0.030** | 0.001 | 0.027 |
|  | Indirect via Type of visit | 0.000 | 0.001 | 0.986 | -0.002 | 0.002 |
|  | Indirect via Tooth brushing frequency | 0.000 | 0.001 | 0.681 | -0.001 | 0.001 |
|  |  |  |  |  |  |  |
| Self rated oral health status | Indirect via Dental visits frequency | -0.004 | 0.002 | **0.034** | -0.007 | 0.000 |
|  | Indirect via Type of visit | 0.000 | 0.000 | 0.986 | -0.001 | 0.001 |
|  | Indirect via Tooth brushing frequency | -0.001 | 0.002 | 0.677 | -0.004 | 0.003 |
|  |  |  |  |  |  |  |
| Insurance –> | | | | | | |
|  |  |  |  |  |  |  |
| Dental visits frequency | Direct | -0.044 | 0.019 | **0.019** | -0.081 | -0.007 |
| Type of visit |  | 0.013 | 0.030 | 0.668 | -0.046 | 0.072 |
| Tooth brushing frequency |  | -0.053 | 0.021 | **0.010** | -0.093 | -0.013 |
| Sweets consumption |  | -0.054 | 0.019 | **0.004** | -0.091 | -0.017 |
| Soft drinks consumption |  | -0.050 | 0.020 | **0.011** | -0.089 | -0.011 |
|  |  |  |  |  |  |  |
| Oral Pain | Indirect via Dental visits frequency | -0.022 | 0.010 | **0.018** | -0.041 | -0.004 |
|  | Indirect via Type of visit | -0.001 | 0.002 | 0.669 | -0.005 | 0.003 |
|  | Indirect via Tooth brushing frequency | 0.002 | 0.001 | **0.041** | 0.000 | 0.004 |
|  | Indirect via Sweets consumption | 0.000 | 0.001 | 0.519 | -0.001 | 0.001 |
|  | Indirect via Soft drinks consumption | -0.001 | 0.001 | 0.115 | -0.002 | 0.000 |
|  |  |  |  |  |  |  |
| Self rated oral health status | Indirect via Dental visits frequency | 0.006 | 0.002 | **0.021** | 0.001 | 0.011 |
|  | Indirect via Type of visit | 0.000 | 0.001 | 0.690 | -0.001 | 0.002 |
|  | Indirect via Tooth brushing frequency | -0.007 | 0.003 | **0.012** | -0.012 | -0.001 |
|  | Indirect via Sweets consumption | 0.005 | 0.002 | **0.009** | 0.001 | 0.008 |
|  | Indirect via Soft drinks consumption | -0.001 | 0.001 | 0.161 | -0.003 | 0.000 |
|  |  |  |  |  |  |  |
| Access to oral healthcare | | | | | | |
|  |  |  |  |  |  |  |
| Dental visits frequency | Direct | 0.394 | 0.009 | **<0.001** | 0.375 | 0.412 |
| Type of visit |  | -0.146 | 0.011 | **<0.001** | -0.168 | -0.123 |
| Tooth brushing frequency |  | -0.123 | 0.010 | **<0.001** | -0.143 | -0.103 |
| Sweets consumption |  | -0.018 | 0.011 | 0.124 | -0.040 | 0.005 |
| Soft drinks consumption |  | 0.009 | 0.011 | 0.443 | -0.014 | 0.031 |
|  |  |  |  |  |  |  |
| Oral Pain | Indirect via Dental visits frequency | 0.201 | 0.006 | **<0.001** | 0.188 | 0.213 |
|  | Indirect via Type of visit | 0.009 | 0.002 | **<0.001** | 0.005 | 0.014 |
|  | Indirect via Tooth brushing frequency | 0.005 | 0.001 | **<0.001** | 0.002 | 0.007 |
|  | Indirect via Sweets consumption | 0.000 | 0.000 | 0.540 | -0.001 | 0.000 |
|  | Indirect via Soft drinks consumption | 0.000 | 0.000 | 0.477 | 0.000 | 0.001 |
|  |  |  |  |  |  |  |
| Self rated oral health status | Indirect via Dental visits frequency | -0.051 | 0.006 | **<0.001** | -0.062 | -0.040 |
|  | Indirect via Type of visit | -0.003 | 0.002 | 0.175 | -0.007 | 0.001 |
|  | Indirect via Tooth brushing frequency | -0.016 | 0.002 | **<0.001** | -0.020 | -0.012 |
|  | Indirect via Sweets consumption | 0.002 | 0.001 | 0.132 | 0.000 | 0.003 |
|  | Indirect via Soft drinks consumption | 0.000 | 0.000 | 0.499 | 0.000 | 0.001 |
|  |  |  |  |  |  |  |
| Source of care | | | | | | |
|  |  |  |  |  |  |  |
| Dental visits frequency | Direct | -0.011 | 0.019 | 0.547 | -0.048 | 0.025 |
| Type of visit |  | 0.074 | 0.025 | **0.004** | 0.024 | 0.124 |
| Tooth brushing frequency |  | 0.129 | 0.021 | **<0.001** | 0.088 | 0.170 |
| Sweets consumption |  | 0.095 | 0.020 | **<0.001** | 0.055 | 0.135 |
| Soft drinks consumption |  | 0.036 | 0.020 | 0.073 | -0.003 | 0.075 |
|  |  |  |  |  |  |  |
| Oral Pain | Indirect via Dental visits frequency | -0.006 | 0.009 | 0.547 | -0.024 | 0.013 |
|  | Indirect via Type of visit | -0.005 | 0.002 | **0.017** | -0.009 | -0.001 |
|  | Indirect via Tooth brushing frequency | -0.005 | 0.002 | **0.003** | -0.008 | -0.002 |
|  | Indirect via Sweets consumption | 0.001 | 0.001 | 0.517 | -0.001 | 0.003 |
|  | Indirect via Soft drinks consumption | 0.001 | 0.001 | 0.183 | 0.000 | 0.002 |
|  |  |  |  |  |  |  |
| Self rated oral health status | Indirect via Dental visits frequency | 0.001 | 0.002 | 0.550 | -0.003 | 0.006 |
|  | Indirect via Type of visit | 0.002 | 0.001 | 0.232 | -0.001 | 0.004 |
|  | Indirect via Tooth brushing frequency | 0.017 | 0.003 | **<0.001** | 0.010 | 0.023 |
|  | Indirect via Sweets consumption | -0.008 | 0.002 | **<0.001** | -0.012 | -0.004 |
|  | Indirect via Soft drinks consumption | 0.001 | 0.001 | 0.241 | -0.001 | 0.002 |

**Table S2. Direct and Indirect Effects on Oral Pain and Self-Rated Oral Health Status in Saudi Arabia for 15–24 years**

|  | Pathway | β | SE | *p*-value | 95% CI | |
| --- | --- | --- | --- | --- | --- | --- |
| Age –> | Direct |  |  |  |  |  |
| Dental visits frequency |  | 0.019 | 0.012 | 0.124 | -0.005 | 0.044 |
| Type of visit |  | -0.032 | 0.016 | **0.043** | -0.063 | -0.001 |
| Tooth brushing frequency |  | -0.011 | 0.013 | 0.405 | -0.036 | 0.015 |
| Sweets consumption |  | -0.008 | 0.012 | 0.527 | -0.032 | 0.016 |
| Soft drinks consumption |  | -0.004 | 0.013 | 0.764 | -0.029 | 0.022 |
|  |  |  |  |  |  |  |
| Age –> Oral Pain | Indirect via Dental visits frequency | 0.012 | 0.008 | 0.124 | -0.003 | 0.027 |
|  | Indirect via Type of visit | 0.002 | 0.001 | **0.047** | 0.000 | 0.004 |
|  | Indirect via Tooth brushing frequency | 0.001 | 0.001 | 0.407 | -0.001 | 0.004 |
|  | Indirect via Sweets consumption | 0.000 | 0.000 | 0.541 | -0.001 | 0.001 |
|  | Indirect via Soft drinks consumption | 0.000 | 0.000 | 0.765 | -0.001 | 0.001 |
|  |  |  |  |  |  |  |
| Age –> Self rated oral health status | Indirect via Dental visits frequency | -0.003 | 0.002 | 0.128 | -0.006 | 0.001 |
|  | Indirect via Type of visit | -0.001 | 0.001 | 0.111 | -0.003 | 0.000 |
|  | Indirect via Tooth brushing frequency | -0.002 | 0.002 | 0.406 | -0.006 | 0.002 |
|  | Indirect via Sweets consumption | 0.001 | 0.001 | 0.528 | -0.001 | 0.003 |
|  | Indirect via Soft drinks consumption | 0.000 | 0.001 | 0.765 | -0.002 | 0.001 |
|  |  |  |  |  |  |  |
| Sex –> | Direct |  |  |  |  |  |
| Dental visits frequency |  | -0.004 | 0.013 | 0.741 | -0.03 | 0.022 |
| Type of visit |  | 0.006 | 0.019 | 0.768 | -0.032 | 0.043 |
| Tooth brushing frequency |  | 0.003 | 0.013 | 0.837 | -0.024 | 0.029 |
| Sweets consumption |  | -0.017 | 0.013 | 0.165 | -0.042 | 0.007 |
| Soft drinks consumption |  | -0.029 | 0.013 | **0.023** | -0.054 | -0.004 |
|  |  |  |  |  |  |  |
| Sex –> Oral Pain | Indirect via Dental visits frequency | -0.003 | 0.008 | 0.741 | -0.019 | 0.013 |
|  | Indirect via Type of visit | 0.000 | 0.001 | 0.767 | -0.003 | 0.002 |
|  | Indirect via Tooth brushing frequency | 0.000 | 0.001 | 0.837 | -0.003 | 0.002 |
|  | Indirect via Sweets consumption | -0.001 | 0.000 | 0.210 | -0.001 | 0.000 |
|  | Indirect via Soft drinks consumption | 0.001 | 0.001 | 0.071 | 0.000 | 0.002 |
|  |  |  |  |  |  |  |
| Sex –> Self rated oral health status | Indirect via Dental visits frequency | 0.001 | 0.002 | 0.741 | -0.003 | 0.004 |
|  | Indirect via Type of visit | 0.000 | 0.001 | 0.767 | -0.001 | 0.002 |
|  | Indirect via Tooth brushing frequency | 0.000 | 0.002 | 0.837 | -0.004 | 0.005 |
|  | Indirect via Sweets consumption | 0.001 | 0.001 | 0.174 | -0.001 | 0.003 |
|  | Indirect via Soft drinks consumption | -0.002 | 0.001 | **0.049** | -0.004 | 0.000 |
|  |  |  |  |  |  |  |
| Citizenship –> | Direct |  |  |  |  |  |
| Dental visits frequency |  | 0.043 | 0.021 | **0.045** | 0.001 | 0.084 |
| Type of visit |  | -0.029 | 0.028 | 0.302 | -0.085 | 0.026 |
| Tooth brushing frequency |  | 0.008 | 0.02 | 0.697 | -0.032 | 0.048 |
| Sweets consumption |  | -0.014 | 0.02 | 0.458 | -0.053 | 0.024 |
| Soft drinks consumption |  | 0.013 | 0.021 | 0.542 | -0.029 | 0.055 |
|  |  |  |  |  |  |  |
| Citizenship –> Oral Pain | Indirect via Dental visits frequency | 0.026 | 0.013 | **0.045** | 0.001 | 0.052 |
|  | Indirect via Type of visit | 0.002 | 0.002 | 0.347 | -0.002 | 0.006 |
|  | Indirect via Tooth brushing frequency | -0.001 | 0.002 | 0.697 | -0.005 | 0.003 |
|  | Indirect via Sweets consumption | 0.000 | 0.001 | 0.468 | -0.002 | 0.001 |
|  | Indirect via Soft drinks consumption | 0.000 | 0.001 | 0.550 | -0.002 | 0.001 |
|  |  |  |  |  |  |  |
| Citizenship –> Self rated oral health status | Indirect via Dental visits frequency | -0.006 | 0.003 | 0.050 | -0.012 | 0.000 |
|  | Indirect via Type of visit | -0.001 | 0.001 | 0.350 | -0.004 | 0.001 |
|  | Indirect via Tooth brushing frequency | 0.001 | 0.003 | 0.698 | -0.005 | 0.008 |
|  | Indirect via Sweets consumption | 0.001 | 0.002 | 0.460 | -0.002 | 0.004 |
|  | Indirect via Soft drinks consumption | 0.001 | 0.001 | 0.552 | -0.002 | 0.004 |
|  |  |  |  |  |  |  |
| Region –> | Direct |  |  |  |  |  |
| Dental visits frequency |  | -0.028 | 0.014 | **0.039** | -0.055 | -0.001 |
| Type of visit |  | -0.02 | 0.021 | 0.345 | -0.062 | 0.022 |
| Tooth brushing frequency |  | -0.062 | 0.013 | **<0.001** | -0.088 | -0.037 |
| Sweets consumption |  | -0.113 | 0.011 | **<0.001** | -0.135 | -0.091 |
| Soft drinks consumption |  | 0.023 | 0.012 | **0.060** | -0.001 | 0.047 |
|  |  |  |  |  |  |  |
| Region –> Oral Pain | Indirect via Dental visits frequency | -0.017 | 0.008 | **0.039** | -0.034 | -0.001 |
|  | Indirect via Type of visit | 0.001 | 0.001 | 0.377 | -0.002 | 0.004 |
|  | Indirect via Tooth brushing frequency | 0.006 | 0.001 | **<0.001** | 0.003 | 0.009 |
|  | Indirect via Sweets consumption | -0.004 | 0.001 | **0.010** | -0.007 | -0.001 |
|  | Indirect via Soft drinks consumption | -0.001 | 0.001 | 0.124 | -0.002 | 0.000 |
|  |  |  |  |  |  |  |
| Region –> Self rated oral health status | Indirect via Dental visits frequency | 0.004 | 0.002 | 0.050 | 0.000 | 0.008 |
|  | Indirect via Type of visit | -0.001 | 0.001 | 0.380 | -0.003 | 0.001 |
|  | Indirect via Tooth brushing frequency | -0.010 | 0.002 | **<0.001** | -0.014 | -0.006 |
|  | Indirect via Sweets consumption | 0.009 | 0.002 | **<0.001** | 0.005 | 0.013 |
|  | Indirect via Soft drinks consumption | 0.001 | 0.001 | 0.087 | 0.000 | 0.003 |
|  |  |  |  |  |  |  |
| Income –> | Direct |  |  |  |  |  |
| Dental visits frequency |  | -0.014 | 0.018 | 0.449 | -0.049 | 0.022 |
| Type of visit |  | -0.026 | 0.025 | 0.295 | -0.075 | 0.023 |
| Tooth brushing frequency |  | -0.006 | 0.018 | 0.761 | -0.041 | 0.03 |
| Sweets consumption |  | -0.004 | 0.018 | 0.823 | -0.04 | 0.031 |
| Soft drinks consumption |  | -0.001 | 0.018 | 0.951 | -0.037 | 0.035 |
|  |  |  |  |  |  |  |
| Income –> Oral Pain | Indirect via Dental visits frequency | -0.008 | 0.011 | 0.450 | -0.030 | 0.013 |
|  | Indirect via Type of visit | 0.002 | 0.002 | 0.318 | -0.002 | 0.005 |
|  | Indirect via Tooth brushing frequency | 0.001 | 0.002 | 0.761 | -0.003 | 0.004 |
|  | Indirect via Sweets consumption | 0.000 | 0.001 | 0.824 | -0.001 | 0.001 |
|  | Indirect via Soft drinks consumption | 0.000 | 0.001 | 0.951 | -0.001 | 0.001 |
|  |  |  |  |  |  |  |
| Income –> Self rated oral health status | Indirect via Dental visits frequency | 0.002 | 0.003 | 0.451 | -0.003 | 0.007 |
|  | Indirect via Type of visit | -0.001 | 0.001 | 0.333 | -0.004 | 0.001 |
|  | Indirect via Tooth brushing frequency | -0.001 | 0.003 | 0.761 | -0.007 | 0.005 |
|  | Indirect via Sweets consumption | 0.000 | 0.001 | 0.823 | -0.003 | 0.003 |
|  | Indirect via Soft drinks consumption | 0.000 | 0.001 | 0.951 | -0.002 | 0.002 |
|  |  |  |  |  |  |  |
| Accident –> | Direct |  |  |  |  |  |
| Dental visits frequency |  | 0.022 | 0.013 | 0.090 | -0.003 | 0.048 |
| Type of visit |  | -0.019 | 0.013 | 0.149 | -0.044 | 0.007 |
| Tooth brushing frequency |  | 0.006 | 0.013 | 0.662 | -0.02 | 0.032 |
|  |  |  |  |  |  |  |
| Accident –> Oral Pain | Indirect via Dental visits frequency | 0.014 | 0.008 | 0.090 | -0.002 | 0.029 |
|  | Indirect via Type of visit | 0.001 | 0.001 | 0.171 | -0.001 | 0.003 |
|  | Indirect via Tooth brushing frequency | -0.001 | 0.001 | 0.662 | -0.003 | 0.002 |
|  |  |  |  |  |  |  |
| Accident –> Self rated oral health status | Indirect via Dental visits frequency | -0.003 | 0.002 | 0.091 | -0.007 | 0.001 |
|  | Indirect via Type of visit | -0.001 | 0.001 | 0.194 | -0.002 | 0.000 |
|  | Indirect via Tooth brushing frequency | 0.001 | 0.002 | 0.663 | -0.003 | 0.005 |
|  |  |  |  |  |  |  |
| Disability –> | Direct |  |  |  |  |  |
| Dental visits frequency |  | -0.003 | 0.013 | 0.846 | -0.029 | 0.024 |
| Type of visit |  | 0.059 | 0.044 | 0.177 | -0.027 | 0.145 |
| Tooth brushing frequency |  | -0.012 | 0.013 | 0.349 | -0.038 | 0.013 |
|  |  |  |  |  |  |  |
| Disability –> Oral Pain | Indirect via Dental visits frequency | -0.002 | 0.008 | 0.846 | -0.018 | 0.014 |
|  | Indirect via Type of visit | -0.004 | 0.003 | 0.206 | -0.010 | 0.002 |
|  | Indirect via Tooth brushing frequency | 0.001 | 0.001 | 0.351 | -0.001 | 0.004 |
|  |  |  |  |  |  |  |
| Disability –> Self rated oral health status | Indirect via Dental visits frequency | 0.000 | 0.002 | 0.846 | -0.003 | 0.004 |
|  | Indirect via Type of visit | 0.003 | 0.002 | 0.171 | -0.001 | 0.007 |
|  | Indirect via Tooth brushing frequency | -0.002 | 0.002 | 0.351 | -0.006 | 0.002 |
|  |  |  |  |  |  |  |
| body Mass Index –> | Direct |  |  |  |  |  |
| Dental visits frequency |  | -0.02 | 0.014 | 0.149 | -0.048 | 0.007 |
| Type of visit |  | -0.003 | 0.018 | 0.854 | -0.039 | 0.033 |
| Tooth brushing frequency |  | 0.019 | 0.014 | 0.156 | -0.007 | 0.046 |
|  |  |  |  |  |  |  |
| body Mass Index –> Oral Pain | Indirect via Dental visits frequency | -0.013 | 0.009 | 0.150 | -0.030 | 0.005 |
|  | Indirect via Type of visit | 0.000 | 0.001 | 0.855 | -0.002 | 0.003 |
|  | Indirect via Tooth brushing frequency | -0.002 | 0.001 | 0.162 | -0.005 | 0.001 |
|  |  |  |  |  |  |  |
| body Mass Index –> Self rated oral health status | Indirect via Dental visits frequency | 0.003 | 0.002 | 0.157 | -0.001 | 0.007 |
|  | Indirect via Type of visit | 0.000 | 0.001 | 0.854 | -0.002 | 0.001 |
|  | Indirect via Tooth brushing frequency | 0.003 | 0.002 | 0.158 | -0.001 | 0.007 |
|  |  |  |  |  |  |  |
| Insurance–> | Direct |  |  |  |  |  |
| Dental visits frequency |  | -0.037 | 0.026 | 0.149 | -0.087 | 0.013 |
| Type of visit |  | 0.022 | 0.035 | 0.521 | -0.046 | 0.090 |
| Tooth brushing frequency |  | 0.010 | 0.023 | 0.654 | -0.034 | 0.055 |
| Sweets consumption |  | -0.092 | 0.022 | **<0.001** | -0.134 | -0.049 |
| Soft drinks consumption |  | -0.124 | 0.021 | **<0.001** | -0.165 | -0.083 |
|  |  |  |  |  |  |  |
| Insurance –> Oral Pain | Indirect via Dental visits frequency | -0.023 | 0.016 | 0.150 | -0.054 | 0.008 |
|  | Indirect via Type of visit | -0.001 | 0.002 | 0.521 | -0.006 | 0.003 |
|  | Indirect via Tooth brushing frequency | -0.001 | 0.002 | 0.656 | -0.005 | 0.003 |
|  | Indirect via Sweets consumption | -0.003 | 0.001 | **0.027** | -0.006 | 0.000 |
|  | Indirect via Soft drinks consumption | 0.004 | 0.002 | **0.011** | 0.001 | 0.008 |
|  |  |  |  |  |  |  |
| Insurance –> Self rated oral health status | Indirect via Dental visits frequency | 0.005 | 0.004 | 0.161 | -0.002 | 0.013 |
|  | Indirect via Type of visit | 0.001 | 0.002 | 0.531 | -0.002 | 0.004 |
|  | Indirect via Tooth brushing frequency | 0.002 | 0.004 | 0.656 | -0.006 | 0.009 |
|  | Indirect via Sweets consumption | 0.007 | 0.002 | **0.002** | 0.003 | 0.012 |
|  | Indirect via Soft drinks consumption | -0.008 | 0.002 | **<0.001** | -0.012 | -0.004 |
|  |  |  |  |  |  |  |
| Access to oral healthcare –> | Direct |  |  |  |  |  |
| Dental visits frequency |  | -0.016 | 0.013 | 0.218 | -0.042 | 0.010 |
| Type of visit |  | -0.011 | 0.015 | 0.469 | -0.040 | 0.018 |
| Tooth brushing frequency |  | 0.023 | 0.013 | 0.085 | -0.003 | 0.049 |
| Sweets consumption |  | 0.038 | 0.012 | **0.002** | 0.014 | 0.062 |
| Soft drinks consumption |  | 0.039 | 0.013 | **0.003** | 0.013 | 0.064 |
|  |  |  |  |  |  |  |
| Access to oral healthcare –> Oral Pain | Indirect via Dental visits frequency | -0.010 | 0.008 | 0.218 | -0.026 | 0.006 |
|  | Indirect via Type of visit | 0.001 | 0.001 | 0.470 | -0.001 | 0.003 |
|  | Indirect via Tooth brushing frequency | -0.002 | 0.001 | 0.087 | -0.005 | 0.000 |
|  | Indirect via Sweets consumption | 0.001 | 0.001 | **0.039** | 0.000 | 0.002 |
|  | Indirect via Soft drinks consumption | -0.001 | 0.001 | **0.045** | -0.003 | 0.000 |
|  |  |  |  |  |  |  |
| Access to oral healthcare - Self rated oral health status | Indirect via Dental visits frequency | 0.002 | 0.002 | 0.222 | -0.001 | 0.006 |
|  | Indirect via Type of visit | 0.000 | 0.001 | 0.495 | -0.002 | 0.001 |
|  | Indirect via Tooth brushing frequency | 0.004 | 0.002 | 0.094 | -0.001 | 0.008 |
|  | Indirect via Sweets consumption | -0.003 | 0.001 | **0.008** | -0.005 | -0.001 |
|  | Indirect via Soft drinks consumption | 0.002 | 0.001 | **0.015** | 0.000 | 0.004 |
|  |  |  |  |  |  |  |
| Source of care –>  Dental visits frequency  Type of visit  Tooth brushing frequency  Sweets consumption  Soft drinks consumption | Direct |  |  |  |  |  |
|  |  | -0.021 | 0.018 | 0.258 | -0.057 | 0.015 |
|  |  | 0.021 | 0.026 | 0.427 | -0.031 | 0.072 |
|  |  | 0.019 | 0.018 | 0.287 | -0.016 | 0.054 |
|  |  | 0.020 | 0.017 | 0.227 | -0.013 | 0.054 |
|  |  | 0.010 | 0.018 | 0.559 | -0.024 | 0.045 |
|  |  |  |  |  |  |  |
| Source of care –> Oral Pain | Indirect via Dental visits frequency | -0.013 | 0.011 | 0.258 | -0.035 | 0.009 |
|  | Indirect via Type of visit | -0.001 | 0.002 | 0.455 | -0.005 | 0.002 |
|  | Indirect via Tooth brushing frequency | -0.002 | 0.002 | 0.291 | -0.005 | 0.002 |
|  | Indirect via Sweets consumption | 0.001 | 0.001 | 0.271 | -0.001 | 0.002 |
|  | Indirect via Soft drinks consumption | 0.000 | 0.001 | 0.566 | -0.002 | 0.001 |
|  |  |  |  |  |  |  |
| Source of care –> Self rated oral health status | Indirect via Dental visits frequency | 0.003 | 0.003 | 0.259 | -0.002 | 0.008 |
|  | Indirect via Type of visit | 0.001 | 0.001 | 0.456 | -0.002 | 0.003 |
|  | Indirect via Tooth brushing frequency | 0.003 | 0.003 | 0.287 | -0.003 | 0.009 |
|  | Indirect via Sweets consumption | -0.002 | 0.001 | 0.237 | -0.004 | 0.001 |
|  | Indirect via Soft drinks consumption | 0.001 | 0.001 | 0.563 | -0.002 | 0.003 |

**Table S3. Direct and Indirect Effects on Oral Pain and Self-Rated Oral Health Status in Saudi Arabia for ≥25 years**

|  | Pathway | β | SE | *p*-value | 95% CI | |
| --- | --- | --- | --- | --- | --- | --- |
| Age –> | Direct |  |  |  |  |  |
| Dental visits frequency |  | 0.000 | 0.012 | 0.982 | -0.023 | 0.023 |
| Type of visit |  | 0.009 | 0.015 | 0.553 | -0.020 | 0.037 |
| Tooth brushing frequency |  | 0.004 | 0.011 | 0.683 | -0.017 | 0.026 |
| Smoking |  | -0.004 | 0.010 | 0.652 | -0.023 | 0.015 |
| Sweets consumption |  | 0.007 | 0.010 | 0.478 | -0.013 | 0.027 |
| Soft drinks consumption |  | 0.008 | 0.010 | 0.427 | -0.012 | 0.029 |
|  |  |  |  |  |  |  |
| Age –> Oral Pain | Indirect via Dental visits frequency | 0.000 | 0.007 | 0.982 | -0.013 | 0.013 |
|  | Indirect via Type of visit | -0.001 | 0.001 | 0.564 | -0.003 | 0.002 |
|  | Indirect via Tooth brushing frequency | 0.000 | 0.001 | 0.684 | -0.002 | 0.001 |
|  | Indirect via Smoking | 0.000 | 0.000 | 0.653 | -0.001 | 0.001 |
|  | Indirect via Sweets consumption | 0.000 | 0.000 | 0.511 | 0.000 | 0.001 |
|  | Indirect via Soft drinks consumption | 0.000 | 0.000 | 0.480 | -0.001 | 0.000 |
|  |  |  |  |  |  |  |
| Age –> Self rated oral health status | Indirect via Dental visits frequency | 0.000 | 0.002 | 0.982 | -0.003 | 0.003 |
|  | Indirect via Type of visit | 0.000 | 0.001 | 0.569 | -0.001 | 0.002 |
|  | Indirect via Tooth brushing frequency | 0.001 | 0.002 | 0.683 | -0.003 | 0.004 |
|  | Indirect via Smoking | 0.000 | 0.000 | 0.683 | 0.000 | 0.000 |
|  | Indirect via Sweets consumption | 0.000 | 0.001 | 0.478 | -0.002 | 0.001 |
|  | Indirect via Soft drinks consumption | 0.001 | 0.001 | 0.430 | -0.001 | 0.002 |
|  |  |  |  |  |  |  |
| Sex –> | Direct |  |  |  |  |  |
| Dental visits frequency |  | -0.027 | 0.013 | **0.037** | -0.052 | -0.002 |
| Type of visit |  | -0.017 | 0.015 | 0.262 | -0.047 | 0.013 |
| Tooth brushing frequency |  | 0.026 | 0.012 | **0.035** | 0.002 | 0.050 |
| Smoking |  | -0.010 | 0.011 | 0.348 | -0.032 | 0.011 |
| Sweets consumption |  | 0.002 | 0.012 | 0.844 | -0.021 | 0.026 |
| Soft drinks consumption |  | -0.005 | 0.012 | 0.695 | -0.027 | 0.018 |
|  |  |  |  |  |  |  |
| Sex –> Oral Pain | Indirect via Dental visits frequency | -0.015 | 0.007 | **0.039** | -0.030 | -0.001 |
|  | Indirect via Type of visit | 0.002 | 0.001 | 0.259 | -0.001 | 0.004 |
|  | Indirect via Tooth brushing frequency | -0.002 | 0.001 | **0.044** | -0.004 | 0.000 |
|  | Indirect via Smoking | 0.000 | 0.001 | 0.374 | -0.001 | 0.001 |
|  | Indirect via Sweets consumption | 0.000 | 0.000 | 0.845 | 0.000 | 0.000 |
|  | Indirect via Soft drinks consumption | 0.000 | 0.000 | 0.700 | 0.000 | 0.001 |
|  |  |  |  |  |  |  |
| Sex –> Self rated oral health status | Indirect via Dental visits frequency | 0.004 | 0.002 | **0.049** | 0.000 | 0.007 |
|  | Indirect via Type of visit | -0.001 | 0.001 | 0.256 | -0.002 | 0.001 |
|  | Indirect via Tooth brushing frequency | 0.004 | 0.002 | **0.038** | 0.000 | 0.008 |
|  | Indirect via Smoking | 0.000 | 0.000 | 0.531 | 0.000 | 0.001 |
|  | Indirect via Sweets consumption | 0.000 | 0.001 | 0.844 | -0.002 | 0.001 |
|  | Indirect via Soft drinks consumption | 0.000 | 0.001 | 0.695 | -0.002 | 0.001 |
|  |  |  |  |  |  |  |
| Citizenship –> | Direct |  |  |  |  |  |
| Dental visits frequency |  | 0.039 | 0.015 | **0.011** | 0.009 | 0.069 |
| Type of visit |  | -0.025 | 0.019 | 0.176 | -0.062 | 0.011 |
| Tooth brushing frequency |  | 0.011 | 0.015 | 0.459 | -0.019 | 0.041 |
| Smoking |  | -0.022 | 0.014 | 0.112 | -0.050 | 0.005 |
| Sweets consumption |  | 0.023 | 0.015 | 0.128 | -0.007 | 0.052 |
| Soft drinks consumption |  | 0.014 | 0.015 | 0.348 | -0.015 | 0.043 |
|  |  |  |  |  |  |  |
| Citizenship –> Oral Pain | Indirect via Dental visits frequency | 0.022 | 0.009 | **0.012** | 0.005 | 0.040 |
|  | Indirect via Type of visit | 0.002 | 0.002 | 0.189 | -0.001 | 0.006 |
|  | Indirect via Tooth brushing frequency | -0.001 | 0.001 | 0.463 | -0.003 | 0.002 |
|  | Indirect via Smoking | -0.001 | 0.001 | 0.122 | -0.002 | 0.000 |
|  | Indirect via Sweets consumption | 0.000 | 0.000 | 0.277 | 0.000 | 0.001 |
|  | Indirect via Soft drinks consumption | 0.000 | 0.000 | 0.413 | -0.001 | 0.000 |
|  |  |  |  |  |  |  |
| Citizenship –> Self rated oral health status | Indirect via Dental visits frequency | -0.005 | 0.002 | **0.021** | -0.009 | -0.001 |
|  | Indirect via Type of visit | -0.001 | 0.001 | 0.211 | -0.003 | 0.001 |
|  | Indirect via Tooth brushing frequency | 0.002 | 0.002 | 0.458 | -0.003 | 0.007 |
|  | Indirect via Smoking | 0.000 | 0.000 | 0.461 | 0.000 | 0.001 |
|  | Indirect via Sweets consumption | -0.001 | 0.001 | 0.158 | -0.004 | 0.001 |
|  | Indirect via Soft drinks consumption | 0.001 | 0.001 | 0.352 | -0.001 | 0.003 |
|  |  |  |  |  |  |  |
| Region –> | Direct |  |  |  |  |  |
| Dental visits frequency |  | -0.022 | 0.013 | 0.099 | -0.048 | 0.004 |
| Type of visit |  | 0.012 | 0.018 | 0.501 | -0.023 | 0.047 |
| Tooth brushing frequency |  | -0.078 | 0.013 | **<0.001** | -0.103 | -0.054 |
| Smoking |  | 0.236 | 0.013 | **<0.001** | 0.210 | 0.262 |
| Sweets consumption |  | -0.125 | 0.010 | **<0.001** | -0.145 | -0.104 |
| Soft drinks consumption |  | 0.011 | 0.011 | 0.334 | -0.011 | 0.032 |
|  |  |  |  |  |  |  |
| Region –> Oral Pain | Indirect via Dental visits frequency | -0.013 | 0.008 | 0.098 | -0.028 | 0.002 |
|  | Indirect via Type of visit | -0.001 | 0.002 | 0.496 | -0.004 | 0.002 |
|  | Indirect via Tooth brushing frequency | 0.006 | 0.001 | **<0.001** | 0.004 | 0.009 |
|  | Indirect via Smoking | 0.011 | 0.003 | **<0.001** | 0.005 | 0.016 |
|  | Indirect via Sweets consumption | -0.002 | 0.001 | 0.107 | -0.005 | 0.001 |
|  | Indirect via Soft drinks consumption | 0.000 | 0.000 | 0.403 | -0.001 | 0.000 |
|  |  |  |  |  |  |  |
| Region –> Self rated oral health status | Indirect via Dental visits frequency | 0.003 | 0.002 | 0.091 | 0.000 | 0.006 |
|  | Indirect via Type of visit | 0.001 | 0.001 | 0.499 | -0.001 | 0.002 |
|  | Indirect via Tooth brushing frequency | -0.012 | 0.002 | **<0.001** | -0.017 | -0.008 |
|  | Indirect via Smoking | -0.003 | 0.004 | 0.403 | -0.010 | 0.004 |
|  | Indirect via Sweets consumption | 0.008 | 0.002 | **<0.001** | 0.004 | 0.012 |
|  | Indirect via Soft drinks consumption | 0.001 | 0.001 | 0.346 | -0.001 | 0.002 |
|  |  |  |  |  |  |  |
| Marital status –> | Direct |  |  |  |  |  |
| Dental visits frequency |  | 0.003 | 0.021 | 0.888 | -0.039 | 0.045 |
| Type of visit |  | 0.043 | 0.027 | 0.109 | -0.010 | 0.097 |
| Tooth brushing frequency |  | -0.036 | 0.019 | 0.056 | -0.072 | 0.001 |
| Smoking |  | 0.053 | 0.023 | **0.023** | 0.007 | 0.098 |
| Sweets consumption |  | -0.007 | 0.018 | 0.683 | -0.043 | 0.028 |
| Soft drinks consumption |  | 0.019 | 0.017 | 0.251 | -0.014 | 0.053 |
|  |  |  |  |  |  |  |
| Marital status –> Oral Pain | Indirect via Dental visits frequency | 0.002 | 0.012 | 0.888 | -0.023 | 0.026 |
|  | Indirect via Type of visit | -0.004 | 0.003 | 0.132 | -0.009 | 0.001 |
|  | Indirect via Tooth brushing frequency | 0.003 | 0.002 | 0.065 | 0.000 | 0.006 |
|  | Indirect via Smoking | 0.002 | 0.001 | 0.059 | 0.000 | 0.005 |
|  | Indirect via Sweets consumption | 0.000 | 0.000 | 0.688 | -0.001 | 0.001 |
|  | Indirect via Soft drinks consumption | 0.000 | 0.000 | 0.309 | -0.001 | 0.000 |
|  |  |  |  |  |  |  |
| Marital status –> Self rated oral health status | Indirect via Dental visits frequency | 0.000 | 0.003 | 0.888 | -0.006 | 0.005 |
|  | Indirect via Type of visit | 0.002 | 0.001 | 0.151 | -0.001 | 0.005 |
|  | Indirect via Tooth brushing frequency | -0.006 | 0.003 | 0.061 | -0.012 | 0.000 |
|  | Indirect via Smoking | -0.001 | 0.001 | 0.434 | -0.002 | 0.001 |
|  | Indirect via Sweets consumption | 0.000 | 0.001 | 0.685 | -0.002 | 0.003 |
|  | Indirect via Soft drinks consumption | 0.001 | 0.001 | 0.268 | -0.001 | 0.004 |
|  |  |  |  |  |  |  |
| Education –> | Direct |  |  |  |  |  |
| Dental visits frequency |  | 0.024 | 0.018 | 0.190 | -0.012 | 0.059 |
| Type of visit |  | 0.008 | 0.018 | 0.672 | -0.028 | 0.044 |
| Tooth brushing frequency |  | 0.053 | 0.016 | **0.001** | 0.023 | 0.084 |
| Smoking |  | 0.016 | 0.015 | 0.275 | -0.013 | 0.045 |
| Sweets consumption |  | 0.024 | 0.015 | 0.106 | -0.005 | 0.052 |
| Soft drinks consumption |  | 0.003 | 0.015 | 0.831 | -0.025 | 0.032 |
|  |  |  |  |  |  |  |
| Education –> Oral Pain | Indirect via Dental visits frequency | 0.014 | 0.010 | 0.190 | -0.007 | 0.034 |
|  | Indirect via Type of visit | -0.001 | 0.002 | 0.670 | -0.004 | 0.002 |
|  | Indirect via Tooth brushing frequency | -0.004 | 0.001 | **0.002** | -0.007 | -0.002 |
|  | Indirect via Smoking | 0.001 | 0.001 | 0.304 | -0.001 | 0.002 |
|  | Indirect via Sweets consumption | 0.000 | 0.000 | 0.253 | 0.000 | 0.001 |
|  | Indirect via Soft drinks consumption | 0.000 | 0.000 | 0.832 | -0.001 | 0.001 |
|  |  |  |  |  |  |  |
| Education –> Self rated oral health status | Indirect via Dental visits frequency | -0.003 | 0.002 | 0.187 | -0.008 | 0.002 |
|  | Indirect via Type of visit | 0.000 | 0.001 | 0.670 | -0.001 | 0.002 |
|  | Indirect via Tooth brushing frequency | 0.008 | 0.003 | **0.001** | 0.003 | 0.014 |
|  | Indirect via Smoking | 0.000 | 0.000 | 0.498 | -0.001 | 0.000 |
|  | Indirect via Sweets consumption | -0.002 | 0.001 | 0.123 | -0.004 | 0.000 |
|  | Indirect via Soft drinks consumption | 0.000 | 0.001 | 0.832 | -0.002 | 0.002 |
|  |  |  |  |  |  |  |
| Income –> | Direct |  |  |  |  |  |
| Dental visits frequency |  | 0.017 | 0.019 | 0.387 | -0.021 | 0.054 |
| Type of visit |  | -0.017 | 0.024 | 0.477 | -0.065 | 0.031 |
| Tooth brushing frequency |  | 0.013 | 0.018 | 0.466 | -0.022 | 0.048 |
| Smoking |  | -0.004 | 0.014 | 0.770 | -0.032 | 0.024 |
| Sweets consumption |  | -0.002 | 0.017 | 0.911 | -0.035 | 0.031 |
| Soft drinks consumption |  | 0.014 | 0.016 | 0.378 | -0.018 | 0.046 |
|  |  |  |  |  |  |  |
| Income –> Oral Pain | Indirect via Dental visits frequency | 0.010 | 0.011 | 0.385 | -0.012 | 0.031 |
|  | Indirect via Type of visit | 0.002 | 0.002 | 0.475 | -0.003 | 0.006 |
|  | Indirect via Tooth brushing frequency | -0.001 | 0.001 | 0.469 | -0.004 | 0.002 |
|  | Indirect via Smoking | 0.000 | 0.001 | 0.771 | -0.001 | 0.001 |
|  | Indirect via Sweets consumption | 0.000 | 0.000 | 0.911 | -0.001 | 0.001 |
|  | Indirect via Soft drinks consumption | 0.000 | 0.000 | 0.428 | -0.001 | 0.000 |
|  |  |  |  |  |  |  |
| Income –> Self rated oral health status | Indirect via Dental visits frequency | -0.002 | 0.002 | 0.382 | -0.007 | 0.003 |
|  | Indirect via Type of visit | -0.001 | 0.001 | 0.466 | -0.003 | 0.001 |
|  | Indirect via Tooth brushing frequency | 0.002 | 0.003 | 0.466 | -0.003 | 0.008 |
|  | Indirect via Smoking | 0.000 | 0.000 | 0.788 | 0.000 | 0.000 |
|  | Indirect via Sweets consumption | 0.000 | 0.001 | 0.911 | -0.002 | 0.002 |
|  | Indirect via Soft drinks consumption | 0.001 | 0.001 | 0.387 | -0.001 | 0.003 |
|  |  |  |  |  |  |  |
| Accident –> | Direct |  |  |  |  |  |
| Dental visits frequency |  | -0.024 | 0.015 | 0.102 | -0.053 | 0.005 |
| Type of visit |  | 0.000 | 0.019 | 0.998 | -0.037 | 0.037 |
| Tooth brushing frequency |  | 0.028 | 0.012 | 0.021 | 0.004 | 0.053 |
|  |  |  |  |  |  |  |
| Accident –> Oral Pain | Indirect via Dental visits frequency | -0.014 | 0.008 | 0.102 | -0.030 | 0.003 |
|  | Indirect via Type of visit | 0.000 | 0.002 | 0.998 | -0.003 | 0.003 |
|  | Indirect via Tooth brushing frequency | -0.002 | 0.001 | **0.028** | -0.004 | 0.000 |
|  |  |  |  |  |  |  |
| Accident –> Self rated oral health status | Indirect via Dental visits frequency | 0.003 | 0.002 | 0.112 | -0.001 | 0.007 |
|  | Indirect via Type of visit | 0.000 | 0.001 | 0.998 | -0.002 | 0.002 |
|  | Indirect via Tooth brushing frequency | 0.005 | 0.002 | **0.024** | 0.001 | 0.008 |
|  |  |  |  |  |  |  |
| Disability –> | Direct |  |  |  |  |  |
| Dental visits frequency |  | 0.010 | 0.018 | 0.586 | -0.025 | 0.044 |
| Type of visit |  | 0.011 | 0.013 | 0.420 | -0.015 | 0.036 |
| Tooth brushing frequency |  | 0.004 | 0.013 | 0.771 | -0.022 | 0.030 |
|  |  |  |  |  |  |  |
| Disability –> Oral Pain | Indirect via Dental visits frequency | 0.006 | 0.010 | 0.586 | -0.014 | 0.025 |
|  | Indirect via Type of visit | -0.001 | 0.001 | 0.428 | -0.003 | 0.001 |
|  | Indirect via Tooth brushing frequency | 0.000 | 0.001 | 0.772 | -0.002 | 0.002 |
|  |  |  |  |  |  |  |
| Disability –> Self rated oral health status | Indirect via Dental visits frequency | -0.001 | 0.002 | 0.587 | -0.006 | 0.003 |
|  | Indirect via Type of visit | 0.000 | 0.001 | 0.443 | -0.001 | 0.002 |
|  | Indirect via Tooth brushing frequency | 0.001 | 0.002 | 0.771 | -0.004 | 0.005 |
|  |  |  |  |  |  |  |
| body Mass Index –> | Direct |  |  |  |  |  |
| Dental visits frequency |  | -0.028 | 0.013 | **0.033** | -0.055 | -0.002 |
| Type of visit |  | 0.047 | 0.018 | **0.007** | 0.013 | 0.082 |
| Tooth brushing frequency |  | -0.011 | 0.013 | 0.385 | -0.037 | 0.014 |
|  |  |  |  |  |  |  |
| body Mass Index –> Oral Pain | Indirect via Dental visits frequency | -0.016 | 0.008 | **0.033** | -0.032 | -0.001 |
|  | Indirect via Type of visit | -0.004 | 0.002 | **0.011** | -0.007 | -0.001 |
|  | Indirect via Tooth brushing frequency | 0.001 | 0.001 | 0.389 | -0.001 | 0.003 |
|  |  |  |  |  |  |  |
| body Mass Index –> Self rated oral health status | Indirect via Dental visits frequency | 0.004 | 0.002 | **0.039** | 0.000 | 0.007 |
|  | Indirect via Type of visit | 0.002 | 0.001 | **0.030** | 0.000 | 0.004 |
|  | Indirect via Tooth brushing frequency | -0.002 | 0.002 | 0.386 | -0.006 | 0.002 |
|  |  |  |  |  |  |  |
| Insurance –> | Direct |  |  |  |  |  |
| Dental visits frequency |  | -0.049 | 0.019 | **0.011** | -0.087 | -0.011 |
| Type of visit |  | -0.009 | 0.020 | 0.670 | -0.048 | 0.031 |
| Tooth brushing frequency |  | -0.086 | 0.018 | **<0.001** | -0.122 | -0.050 |
| Smoking |  | 0.003 | 0.017 | 0.881 | -0.031 | 0.036 |
| Sweets consumption |  | -0.172 | 0.018 | **<0.001** | -0.207 | -0.138 |
| Soft drinks consumption |  | -0.227 | 0.017 | **<0.001** | -0.260 | -0.193 |
|  |  |  |  |  |  |  |
| Insurance –> Oral Pain | Indirect via Dental visits frequency | -0.028 | 0.011 | **0.011** | -0.050 | -0.007 |
|  | Indirect via Type of visit | 0.001 | 0.002 | 0.671 | -0.003 | 0.004 |
|  | Indirect via Tooth brushing frequency | 0.007 | 0.002 | **<0.001** | 0.003 | 0.010 |
|  | Indirect via Smoking | 0.000 | 0.001 | 0.881 | -0.001 | 0.002 |
|  | Indirect via Sweets consumption | -0.003 | 0.002 | 0.111 | -0.007 | 0.001 |
|  | Indirect via Soft drinks consumption | 0.005 | 0.003 | 0.065 | 0.000 | 0.011 |
|  |  |  |  |  |  |  |
| Insurance –> Self rated oral health status | Indirect via Dental visits frequency | 0.006 | 0.003 | **0.013** | 0.001 | 0.012 |
|  | Indirect via Type of visit | 0.000 | 0.001 | 0.671 | -0.002 | 0.001 |
|  | Indirect via Tooth brushing frequency | -0.014 | 0.003 | **<0.001** | -0.020 | -0.008 |
|  | Indirect via Smoking | 0.000 | 0.000 | 0.882 | 0.000 | 0.000 |
|  | Indirect via Sweets consumption | 0.011 | 0.003 | **<0.001** | 0.006 | 0.017 |
|  | Indirect via Soft drinks consumption | -0.015 | 0.003 | **<0.001** | -0.022 | -0.009 |
|  |  |  |  |  |  |  |
| Access to oral healthcare –> |  |  |  |  |  |  |
| Dental visits frequency | Direct | -0.009 | 0.011 | 0.391 | -0.030 | 0.012 |
| Type of visit |  | 0.011 | 0.017 | 0.515 | -0.022 | 0.045 |
| Tooth brushing frequency |  | 0.044 | 0.011 | **<0.001** | 0.022 | 0.067 |
| Smoking |  | 0.004 | 0.011 | 0.713 | -0.018 | 0.026 |
| Sweets consumption |  | 0.049 | 0.012 | **<0.001** | 0.026 | 0.071 |
| Soft drinks consumption |  | 0.046 | 0.011 | **<0.001** | 0.024 | 0.068 |
|  |  |  |  |  |  |  |
| Access to oral healthcare –> Oral Pain | Indirect via Dental visits frequency | -0.005 | 0.006 | 0.392 | -0.017 | 0.007 |
|  | Indirect via Type of visit | -0.001 | 0.001 | 0.505 | -0.004 | 0.002 |
|  | Indirect via Tooth brushing frequency | -0.004 | 0.001 | **<0.001** | -0.006 | -0.002 |
|  | Indirect via Smoking | 0.000 | 0.001 | 0.717 | -0.001 | 0.001 |
|  | Indirect via Sweets consumption | 0.001 | 0.001 | 0.126 | 0.000 | 0.002 |
|  | Indirect via Soft drinks consumption | -0.001 | 0.001 | 0.097 | -0.002 | 0.000 |
|  |  |  |  |  |  |  |
| Access to oral healthcare –> Self rated oral health status | Indirect via Dental visits frequency | 0.001 | 0.001 | 0.394 | -0.002 | 0.004 |
|  | Indirect via Type of visit | 0.001 | 0.001 | 0.509 | -0.001 | 0.002 |
|  | Indirect via Tooth brushing frequency | 0.007 | 0.002 | **<0.001** | 0.003 | 0.011 |
|  | Indirect via Smoking | 0.000 | 0.000 | 0.758 | 0.000 | 0.000 |
|  | Indirect via Sweets consumption | -0.003 | 0.001 | **0.001** | -0.005 | -0.001 |
|  | Indirect via Soft drinks consumption | 0.003 | 0.001 | **0.001** | 0.001 | 0.005 |
|  |  |  |  |  |  |  |
| Source of care –> | Direct |  |  |  |  |  |
| Dental visits frequency |  | -0.029 | 0.020 | 0.138 | -0.068 | 0.009 |
| Type of visit |  | -0.012 | 0.019 | 0.517 | -0.048 | 0.024 |
| Tooth brushing frequency |  | 0.026 | 0.018 | 0.154 | -0.010 | 0.062 |
| Smoking |  | 0.020 | 0.014 | 0.168 | -0.008 | 0.048 |
| Sweets consumption |  | 0.009 | 0.018 | 0.622 | -0.026 | 0.043 |
| Soft drinks consumption |  | 0.046 | 0.017 | **0.006** | 0.013 | 0.079 |
|  |  |  |  |  |  |  |
| Source of care –> Oral Pain | Indirect via Dental visits frequency | -0.017 | 0.011 | 0.140 | -0.039 | 0.006 |
|  | Indirect via Type of visit | 0.001 | 0.002 | 0.508 | -0.002 | 0.004 |
|  | Indirect via Tooth brushing frequency | -0.002 | 0.001 | 0.158 | -0.005 | 0.001 |
|  | Indirect via Smoking | 0.001 | 0.001 | 0.194 | 0.000 | 0.002 |
|  | Indirect via Sweets consumption | 0.000 | 0.000 | 0.637 | -0.001 | 0.001 |
|  | Indirect via Soft drinks consumption | -0.001 | 0.001 | 0.115 | -0.002 | 0.000 |
|  |  |  |  |  |  |  |
| Source of care –> Self rated oral health status | Indirect via Dental visits frequency | 0.004 | 0.003 | 0.154 | -0.001 | 0.009 |
|  | Indirect via Type of visit | -0.001 | 0.001 | 0.513 | -0.002 | 0.001 |
|  | Indirect via Tooth brushing frequency | 0.004 | 0.003 | 0.159 | -0.002 | 0.010 |
|  | Indirect via Smoking | 0.000 | 0.000 | 0.478 | -0.001 | 0.000 |
|  | Indirect via Sweets consumption | -0.001 | 0.001 | 0.625 | -0.003 | 0.002 |
|  | Indirect via Soft drinks consumption | 0.003 | 0.001 | **0.018** | 0.001 | 0.006 |
